# Genome-wide pervasiveness and localized variation of $k$-mer-based genomic signatures in eukaryotes

**DOI:** 10.1038/s41598-026-40591-7

**Published:** 2026-06-12

**Authors:** Niousha Sadjadi, Camila P. E. de Souza, Gurjit S. Randhawa, Kathleen A. Hill, Lila Kari

**Affiliations:** 1 School of Computer Science, University of Waterloo, Waterloo, ON N2L 3G1 Canada; 2 Department of Statistical and Actuarial Sciences, University of Western Ontario, London, ON N6A 5B7 Canada; 3 School of Computer Science, University of Guelph, Guelph, ON N1G 2W1 Canada; 4 Department of Biology, University of Western Ontario, London, ON N6A 5B7 Canada

**Keywords:** Genomic signature, Chaos game representation (CGR), Frequency chaos game representation (FCGR), Distance measures, Representative sequence, Alignment-free DNA sequence comparison, Computational biology and bioinformatics, Computer science

## Abstract

Genomic signatures–taxon-specific patterns in nucleotide composition–are widely used for taxonomic assignment and comparative genomics, yet their genome-wide pervasiveness across Telomere-to-Telomere assemblies, particularly within functionally diverse and highly repetitive regions, remains undercharacterized. We address this gap with an alignment-free, $k$-mer-based analysis using Frequency Chaos Game Representations (FCGRs) across the human genome and three additional eukaryotes from distinct kingdoms. First, by combining qualitative inspection of FCGR landscapes with quantitative distance benchmarking, we show that each species exhibits a stable genomic signature across most chromosomes, with localized departures concentrated in regions enriched for short and long tandem repeats. Then, we introduce two computational pipelines that automatically select a short, contiguous representative genomic segment (500 Kbp) per genome and use it as a proxy to quantify intragenomic variation. Using DSSIM on a [0,1] scale, 80% of 500 Kbp segments in the human genome lie within 0.24 of the representative; segments exceeding this threshold align with tandem-repeat-dense loci. Leveraging these representatives in downstream tasks yields practical gains–for example, one-nearest-neighbor taxonomic classification improves by 7% relative to choosing a random segment. Finally, we provide k*CGR-Diff*, a graphical tool that enables side-by-side visualization and quantitative comparison of FCGR-based genomic signatures for sample or user-provided sequences, facilitating exploratory analyses of intragenomic variation within and across species. Collectively, our results provide extensive qualitative and quantitative evidence that $k$-mer-based genomic signatures are pervasive at genome scale while varying predictably in repeat-dense regions, and they introduce practical methods and software for proxy selection and comparative analysis.

## Introduction

Recent advances in long-read sequencing and sequence assembly algorithms have led to many Telomere-to-Telomere (T2T) genome sequence assemblies for various species^1,2^, including human, uncovering significant heterogeneity and structural variation within the genome^2^. While it is known that across the genome there are several structural elements (e.g., telomeres, centromeres, and chromosome arms) which differ in function and composition^3^, analysis of T2T sequences has revealed additional insights. Beyond the structural variation that exists within the genome, several factors vary along the length of chromosomes, including GC content, gene density, and the distribution of long interspersed repeat elements (LINEs), long terminal repeats (LTRs), microsatellites, and other repeats^1,3,4^. Furthermore, some studies have shown that even regions traditionally considered highly uniform in sequence composition (e.g., Internal Transcribed Spacers (ITS) and rDNA) exhibit extensive variation across different parts of the genome^5,6^.

In spite of this regional diversity in genome sequence composition associated with local functional attributes, some studies have suggested the presence of certain global characteristics (e.g., dinucleotide relative abundance^7–9^, or the relative frequency of oligonucleotides^10^) that are repeated across different segments of a genome and are referred to as the genomic signature^7^. The studies introducing these concepts hypothesized that the genomic signature is pervasive genome-wide and species-specific, although this conclusion was based on limited analyses of short genomic regions (50 kbp to 100 kbp)^7–9^ or a small number of species^10^. In spite of the small scale of these studies, the assumption of the pervasiveness of genomic signatures within a genome took hold and has been successfully used in numerous applications, including phylogenetics and evolutionary analyses^11,12^, taxonomic classification^10,13,14^, genome adaptation studies^15^, the identification of emergent pathogens^16^, and the classification of cancer genomes^17^.

Prior work has identified various quantitative approaches that could serve as a genomic signature^18–20^. One of the earliest approaches was the use of Dinucleotide Relative Abundance Profiles (DRAPs)^7,8,21–23^, which calculates the ratio of the observed frequency of a dinucleotide (a pair of consecutive nucleotides) to its expected frequency (i.e., the product of the frequencies of its constituent nucleotides). The DRAP concept was then generalized to the oligonucleotide relative abundance profile of a DNA segment, computed as the ratio of the observed frequency of an oligonucleotide to its expected frequency^18^. The Generalized Genomic Signature^24^ is a similar approach that operates by first filtering out the background nucleotide composition and then measuring only the deviation of oligonucleotide frequencies from this background composition.

Another approach to construct a genomic signature is through the Chaos Game Representation (CGR)^25^ of DNA sequences and its derivative, Frequency Chaos Game Representation (FCGR)^26,27^. The CGR of a DNA sequence is a two-dimensional binary image whereby each pixel represents the presence/absence of an oligonucleotide in the sequence^13^, and where the resolution of the image determines the oligonucleotide length^20^. Thus, the CGR of a DNA sequence is a simultaneous representation of the distribution of oligonucleotides of a certain length within that sequence. FCGR generalizes CGR by providing a quantitative view: In an FCGR of resolution $2^k \times 2^k$, where $k$ corresponds to the length of the oligonucleotide^18^ (also referred to as a *k*-mer), the intensity of each pixel represents the frequency of a specific $k$-mer within the sequence, making the entire plot a comprehensive visual representation of $k$-mer frequencies in the originating DNA sequence. FCGRs of DNA sequences exhibit fractal geometric patterns, and the genomic signature represented by an FCGR is correlated to other methods such as DRAP, in that DRAP can be deduced from FCGR^18,20^, but not vice versa^18^. CGR and FCGR have gained significant attention through their usability in bioinformatics^11,28^, due to their ability to visually encapsulate genome-wide sequence composition patterns, and given their robustness, flexibility, and applicability to sequences of any length^10^. Specifically, FCGR has been effectively used for alignment-free genome comparisons in taxonomic analysis^29–31^, thanks to the computational efficiency that results from its alignment-free nature, which allows bypassing the computationally expensive step of multiple sequence alignment^29^.

While the aforementioned studies have suggested that a $k$-mer-based genomic signature is pervasive across the genome of an organism^8,11,12^ and this assumption has been successfully used in various bioinformatics applications, the extent to which this pervasiveness holds throughout a T2T genome assembly remains counterintuitive^12^ and underexplored. In particular, the intragenomic variability of such a genomic signature across different genomic regions still awaits a comprehensive investigation. Should the hypothesis of genomic signature pervasiveness be conclusively proven, an alignment-free genome comparison algorithm would still depend on a method to reliably select a DNA genomic segment that reflects the nucleotide composition characteristics of the whole genome. Given the observed heterogeneity and structural variations within a genome and the expected impact of this heterogeneity on the genomic signature, finding such a representative DNA genomic segment could be challenging.

This study aims to fill these gaps by providing extensive qualitative and quantitative analyses supporting the hypothesis that a $k$-mer-based genomic signature is largely preserved along the length of each individual T2T genome in human and other eukaryotic species, with notable exceptions and localized variations. It also proposes two computational pipelines for selecting a short representative DNA segment that captures the nucleotide composition of the entire genome, at a given resolution ($k$-mer size), and that can be used to explore the sequence composition variability along a T2T assembly. Finally, through several computational experiments, this study demonstrates that short representative genomic segments can be successfully used in downstream tasks such as taxonomic classification. While this initial work focuses on representative model species from different taxonomic groups, the framework is general and can be extended to a broader and more heterogeneous set of genomes.

Concretely, to extract genomic signatures that reliably encapsulate the characteristics of the genome, this study uses FCGR, which allows both visual and quantitative comparisons of genomic signatures. Visual inspection of FCGR images from each chromosome of a T2T genome reveals consistent overall patterns, although variations in image intensity are observed. Beyond a qualitative analysis, this study shows that when comparing FCGRs quantitatively, some distance measures outperform others in capturing notable biological differences in the genomic signature. Through various intra- and intergenomic experiments, the Structural Dissimilarity Index (DSSIM) is suggested as a suitable measure for FCGR comparison for the datasets in this paper.

Using DSSIM, the *Representative Segment Selection Pipeline* (RepSeg) is then introduced as an algorithm to identify a genomic segment whose FCGR has the minimum average distance to those of other segments within the same genome. To increase the computational efficiency of RepSeg, a computationally optimized pipeline, the *Approximate Representative Segment Selection Pipeline* (aRepSeg) is also proposed: Depending on the application, RepSeg can be used when high accuracy is prioritized, while aRepSeg can be used for datasets with large genomes or for time-sensitive applications. The representative segment identified by either pipeline encapsulates genome-wide nucleotide compositions and enables quantitative investigation of intragenomic variation by measuring the distances between constituent genome segments and the representative segment, thereby revealing longitudinal changes in the genomic signature. Also, the representative segment serves as an effective proxy for downstream tasks such as taxonomic classification, and in this study, a benchmark dataset is designed to demonstrate that using representative segments as training samples improves the performance of a one-nearest-neighbor (1-NN) classifier by 7%.

Finally, to facilitate both visual and quantitative comparisons of FCGRs derived from different genomic sequences, and to support the replication of the analysis in this study, a software tool with a graphical user interface (GUI), called *CGR-Diff* is developed. Unlike existing general-purpose GUI tools for intragenomic analysis, such as Integrative Genomics Viewer (IGV)^32^, UGENE^33^, VDAP-GUI^34^, and GEMINI^35^, or alignment-free $k$-mer-frequency comparison tools such as KAST^36^ and TreeWave^37^, *CGR-Diff* is specialized for FCGR-based analysis of the intragenomic variation. The software visualizes the FCGRs of two independent sequences, whether from the same species or different ones, highlights their differences, and provides several quantitative methods to measure their dissimilarity.

The main contributions of this study are:

- Extensive qualitative and quantitative evidence of the as-yet undemonstrated pervasiveness and localized variation of a $k$-mer-based genomic signature in eukaryotes;
- Design and implementation of two computational pipelines that select a contiguous 500 Kbp representative DNA segment to serve as a genome proxy, thereby improving alignment-free taxonomic classification and supporting intragenomic variation analyses. Applying these pipelines to the human genome, we quantified intragenomic variability and found that 80% of 500 Kbp genome segments lie within distance $\textrm{DSSIM}<0.24$ ([0,1] scale) of the representative, indicating strong genome-wide pervasiveness of the signature;
- Development of *CGR-Diff*, a novel software tool with a graphical user interface (GUI) for the visual and quantitative comparisons of FCGR-based genomic signatures of sample or user-provided DNA sequences.

To the best of our knowledge, this is the first study to examine the pervasiveness of FCGR-based genomic signatures across whole genomes of eukaryotic species from four different kingdoms of life, and propose pipelines for selecting a representative DNA segment that preserves the key characteristics of the whole genome.

## Methods

**Fig. 1:**
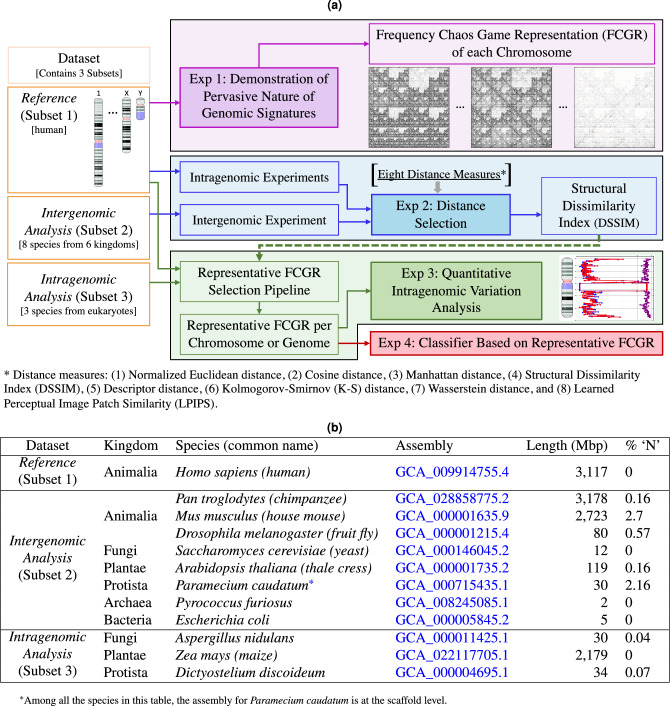
**Method overview and dataset.** (**a**) Overview of the four experiments and their interrelationships: Experiment 1 is an independent study exploring the pervasiveness of genomic signatures across chromosomes within a single species. Experiment 2 conducts internal tests to identify the most appropriate distance measure for comparing genomic signatures. Experiment 3 applies the selected distance measure from Experiment 2 to analyze intragenomic variation across the entire genome through representative segment selection. Experiment 4 assesses the effectiveness of the representative segments suggested by pipelines using a 1-NN classifier. (**b**) Summary of selected species: This includes a list of the selected species for our subsets, detailing their GenBank assemblies from the NCBI database, genome lengths, and the percentage of unknown nucleotides (represented as ‘N’) in their genomes.

This section first describes the dataset and genome sequences utilized in this study. Next, it provides an overview of FCGR, a graphical representation of genomic signatures used in this paper. This is followed by a detailed description of various distance measures employed to compare FCGRs, which serve as a key component of the proposed pipeline. Then, RepSeg is introduced as a pipeline designed to select a short-length representative of the entire genome that can act as a proxy of that genome for computational analyses. To further enhance computational efficiency and reduce memory usage, aRepSeg, an optimized version of RepSeg, is proposed to select representative genome segments more efficiently. Subsequently, *CGR-Diff*, a novel graphical software developed in Python, is described as a tool that enables the visualization and comparison of genomic FCGRs and facilitates both intragenomic and intergenomic studies. The software allows users to either upload two genomic FASTA files or select segments from predefined genomic assemblies of various species, as listed in Fig. 1b. After uploading or selecting sequences, users can identify and select specific segments from each sequence for FCGR comparison. Additionally, *CGR-Diff* offers built-in tests for analyzing intersegment variation, increasing its applicability in genomic research. Finally, the experiments conducted in this study are outlined (see Fig. 1 for an overview of the experiments and dataset).

### Dataset

The DNA sequence dataset consists of three subsets covering the whole genomes of several model species from different kingdoms creating a comprehensive resource for various experiments. The human genome, functioning as the *Reference Subset* (Subset 1), serves as the core and primary data in the study. This choice is justified by the structural and functional complexities of the human genome, along with the extensive annotations and supplementary information available for its Telomere-to-Telomere (T2T) assembly^2,3^. In addition to the human genome, the dataset includes the whole genomes of eleven other species, organized into two different subsets: the *Intergenomic Analysis Subset* (Subset 2), and the *Intragenomic Analysis Subset* (Subset 3). These genomes are specifically selected to be used alongside the human genome for specific parts of the experiments and analyses. The subsequent paragraphs provide details of these genomes, and indicate the source to retrieve their data as well as annotations, in cases where the annotations are available. Additionally, our GitHub page includes the data assembly files for each species, instructions on how to retrieve annotations, and the final annotation files.

To retrieve the human genome, the latest assembly, “T2T-CHM13v2.0,” released in 2022^2,3^, was downloaded from the NCBI database (link in Fig. 1b) on May 9, 2023. This fully annotated assembly, developed by the Telomere-to-Telomere (T2T) consortium^38^, offers a gapless and complete representation of the human genome, covering all chromosomal regions. It contains all known structural variations, including tandem repeats for each chromosome, and features a fully colored and annotated ideogram. The annotations for the different parts of the chromosomes are obtained from GitHub, the NCBI database, or are manually extracted from the NCBI Genome Data Viewer^39^ on Dec 4, 2023.

The *Intergenomic Analysis Subset* (Subset 2) includes the genomes of eight species with different phylogenetic or evolutionary distances from the human genome, which are used for the distance selection experiment. Two of these species, *Pan troglodytes* and *Mus musculus*, are selected from Class Mammalia due to their common origin from an ancestral eutherian genome, which results in strong resemblance in chromosome structures and gene sequences^40^. In addition to the mammalian genomes, six other species are selected for Subset 2, each representing one of the six kingdoms of life: *Drosophila melanogaster* (Animalia), *Saccharomyces cerevisiae* (Fungi), *Arabidopsis thaliana* (Plantae), *Paramecium caudatum* (Protista), *Pyrococcus furiosus* (Archaea), and *Escherichia coli* (Bacteria). The genomes of these species differ significantly from the human genome in terms of karyotypes and gene density. Notably, *Pyrococcus furiosus* (Archaea) and *Escherichia coli* (Bacteria) are prokaryotes, meaning their DNA is not encapsulated within a nucleus, and the compactness and regulatory mechanisms of their genomes differ substantially from those in eukaryotes. These eight species are chosen to span a range of genomic resemblance to the human genome, enabling the evaluation of whether these differences are reflected in the FCGR distance analysis. The assemblies for these species were retrieved on May 25, 2024 from the NCBI database (link in Fig. 1b). Among them, the assemblies of *Saccharomyces cerevisiae* (Fungi), *Pyrococcus furiosus* (Archaea), and *Escherichia coli* (Bacteria) are gapless and complete, containing no unknown nucleotides (‘N’), while the rest of the assemblies include ‘N’ within their genome. In generating FCGRs, these unknown nucleotides are removed without introducing $k$-mer artifacts by discarding $k$-mers that contain ‘N.’ Moreover, among these eight species, all assemblies are at the chromosome level, except for *Paramecium caudatum*, whose assembly is at the scaffold level. Figure 1b includes the additional information of these species including their assembly number in the NCBI database, length of their genome sequence, and the percentage of unknown nucleotides in their assembly.

The *Intragenomic Analysis Subset* (Subset 3) is designed for use alongside the human genome in experiments focused on genome representative segment selection. It includes the genomes of *Aspergillus nidulans* (Fungi), *Zea mays* (Plantae), and *Dictyostelium discoideum* (Protista). The rationale behind the selection of these species is that, first, the species in this subset are eukaryotes with genome lengths comparable to that of the human genome, enabling similar testing of strategies for representative segment selection. Second, each assembly in this subset is chosen from a different kingdom to evaluate the effectiveness of the analysis and assess the generalizability of findings from the human genome to other eukaryotes. The assemblies in Subset 3 were downloaded from the NCBI database (link in Fig. 1b) on Aug 28, 2024, with additional details and annotations for the maize genome extracted from Hufford et al.^41^. The additional information about the species in this subset is included in Fig. 1b.

### Frequency chaos game representation (FCGR)

**Fig. 2:**
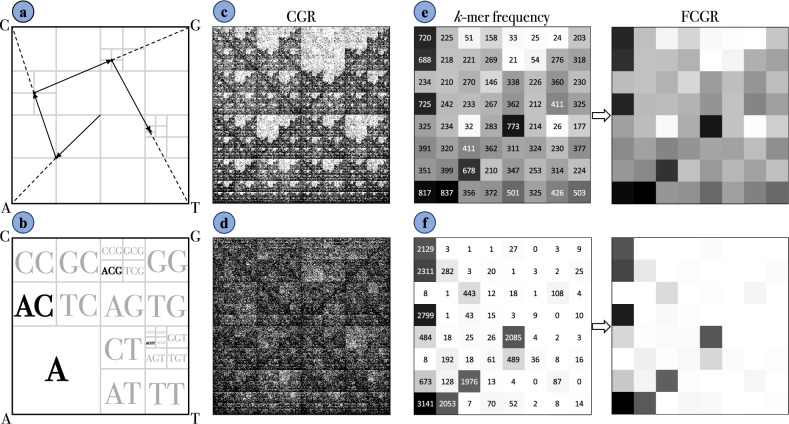
**CGR/FCGR image generation.** (**a**) A schematic of CGR image generation from a DNA sequence. (**b**) Mapping of $k$-mers to specific positions in the CGR. (**c**,**d**) Examples of CGR images ($512 \times 512$, $k=9$) for human (**c**) and maize (**d**). (**e**,**f**) Generating FCGR images by counting $k$-mer frequencies ($k=3$) for human and maize, respectively. (This figure is adapted from Löchel et al.^11^).

Chaos Game Representation (CGR) is a technique to visualize genomic sequences, such as one-dimensional DNA sequences, into two-dimensional visual representations that reflect sequence composition^25^. DNA sequences, composed of four fundamental nucleotide bases–Adenine (A), Cytosine (C), Guanine (G), and Thymine (T)–can be visualized using CGR. To create a CGR image from a sequence, each corner of a square is labeled with one nucleotide. In this analysis, the bottom left corner is labeled A, the top left corner is labeled C, the top right corner is labeled G, and the bottom right corner is labeled T, an arrangement that corresponds to the purine vs. pyrimidine grouping with diagonally opposite corners assigned to purines (A, G) and pyrimidines (C, T)^42^. The center of the square is the starting point, and from there, each nucleotide adds a point to the image, placed halfway between the current point and the corner labeled by that nucleotide^25^ (see Fig. 2a). The final CGR image is a two-dimensional plot reflecting the fractal pattern in the DNA sequence composition. Intuitively, for a predetermined $k$ value, CGR generates a binary image with size $2^k \times 2^k$, where each plotted point corresponds to the presence of a specific $k$-mer in the sequence (see Fig. 2b). Thus, this technique can be considered both a visualization method and a feature extraction method that encodes the distribution of $k$-mers within a DNA sequence (see Fig. 2c,d for the examples of CGR images).

The Frequency Chaos Game Representation (FCGR) is an extension of CGR that quantifies the distribution of points generated by CGR^26^. While CGR creates a fractal image of the sequence, FCGR transforms this visual information into a numerical matrix. The unit square is divided into a grid of resolution $2^k \times 2^k$, where the intensity of each cell corresponds to the frequency of the $k$-mer within the DNA sequence (see Fig. 2e,f). The key difference between CGR and FCGR is that CGR displays $k$-mer abundances and biases in $k$-mer composition, while FCGR provides a quantitative representation of the frequency of $k$-mers of a specific value of $k$ within the sequence. It is noteworthy to mention that some sequences may contain unknown nucleotides, represented as ‘N.’ During FCGR generation, rather than removing these ‘N’s before extracting the $k$-mers, all $k$-mers are first extracted, and then any containing ‘N’ are discarded. This approach prevents the introduction of unwanted $k$-mers that could result from removing ‘N’ directly from the original sequence.

In generating FCGR images, both the size of $k$ and the sequence length influence the visibility of geometric patterns. For a DNA sequence of fixed length, a very small $k$ results in low resolution FCGRs, which provide limited detail and make geometric patterns indistinct. Conversely, a very large $k$ leads to sparsity in $k$-mer frequencies due to the exponential increase in potential $k$-mers, making the geometric patterns within the FCGR image harder to discern. Similarly, when the image resolution is fixed (i.e., $k$ is fixed), shorter sequences contain fewer $k$-mer frequencies, resulting in sparser FCGRs and less discernible geometric patterns. Therefore, an optimal $k$ value must be selected for each sequence length to balance high resolution with a sufficient distribution of $k$-mer frequencies, ensuring that the FCGR remains informative and the geometric patterns are clearly visible.

Figure 2c depicts the CGR image of the human chromosome 21 and Fig. 2d shows the CGR of the maize chromosome 8. Their corresponding FCGR representations for $k=3$ are displayed in Fig. 2e for human and Fig. 2f for maize. As seen in the figure, the CGR/FCGR representations differ significantly between the human and maize chromosomes. Generally, FCGRs have been found to be species-specific, such that CGR/FCGR images of DNA sequences from the same genome are quantitatively more similar, while those from different species’ genomes show considerable differences^13^. Therefore, FCGR images have been successfully used in numerous studies for species identification^27,29,43,44^, taxonomic classification^30,31^ and phylogenetic clustering^12^.

### Distance measures

Distance measures in this study refer to quantified measures of dissimilarity between FCGR images. Several studies have investigated various methods of comparing two FCGR images^11,13,26,45^. Among the existing methods for measuring the dissimilarity of two FCGR images, eight different distance measures are considered: Normalized Euclidean distance, Cosine distance, Manhattan distance, Structural Dissimilarity Index (DSSIM)^46^, Descriptor distance^47^, Kolmogorov-Smirnov (K-S) distance^48^, Wasserstein distance^49^, and Learned Perceptual Image Patch (Dis)Similarity (LPIPS)^50^. Each measure provides a unique way to quantify the dissimilarity between FCGR representations. The Euclidean distance has been widely used in FCGR comparison in different studies^13,27,30^. In order to improve the interpretability of the Euclidean distance values, the Normalized Euclidean distance is used, which limits the upper bound of the values. Manhattan, DSSIM, and Descriptor are included as these performed better than the other distances investigated by Karamichalis et al.^13^ in a study of intergenomic and intragenomic variation. In addition to well-established distance measures in the literature, four additional distances are incorporated. Cosine distance is included for its interpretability and proven effectiveness in machine learning applications^51,52^. The K-S statistic and Wasserstein distance are selected as probability-based distance measures, offering different and unique comparison methods with respect to similar studies. Lastly, LPIPS is chosen as a deep learning-based method that combines both structural and perceptual components for measuring dissimilarity. Details on the calculation of each distance measure and the required preprocessing steps are presented in the Supplementary Material Section A.1.

### Representative segment selection pipeline (RepSeg)

Building on the evidence in this study, demonstrating the pervasive nature of genomic signatures across chromosomes and entire genomes within a species, the Representative Segment Selection Pipeline (RepSeg) is proposed. This pipeline identifies a DNA segment that is significantly shorter than the entire chromosome or genome (e.g., approximately 0.5% of the length of a human chromosome) yet encapsulates the main $k$-mer frequency characteristics of that genome, and closely resembles in this respect most other segments of the same chromosome or genome (hereafter referred to as ‘genome’ for simplicity). The representative segment thus serves as an ideal reference for analyzing intragenomic variations of the genomic signature. Most importantly, the representative segment can act effectively as a proxy of the genome for important applications such as taxonomic classification. This section describes the details of the RepSeg step by step.

The RepSeg begins by dividing a genome into consecutive, non-overlapping segments of equal length. Then, for each segment, it converts the DNA sequence to an FCGR image using a pre-determined $k$-mer value. In the next step, it computes the distance matrix $D$ by calculating the distance between all pairs of FCGRs obtained from the segments. The total number of non-overlapping consecutive segments ($S$) determines the dimensions of the resulting $S \times S$ symmetric distance matrix $D$. Finally, it selects the medoid^53^ (i.e., the segment that minimizes the average of distances to all other segments in the matrix) and designates it as the representative segment for the genome.

**Fig. 3:**
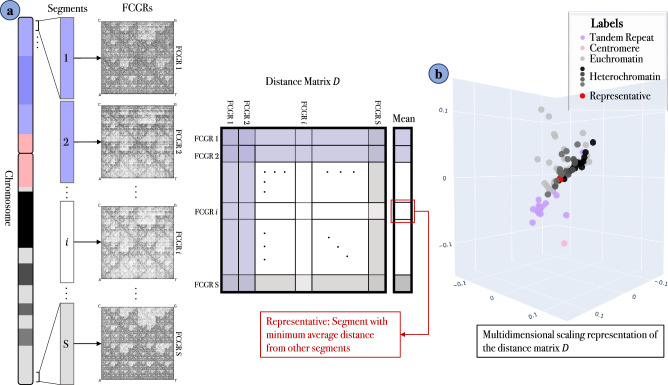
**Summary of the Representative Segment Selection Pipeline (RepSeg).** (**a**) Outlines the steps involved in the pipeline for a chromosome, including chromosome segmentation (e.g., size 500 Kbp), FCGR generation (e.g., using $k=9$), distance matrix calculation, and representative segment selection. (**b**) Multidimensional Scaling (MDS) representation of the distance matrix $D$. The representative segment is highlighted as a red point, representing the center of mass of the MDS plot due to its minimal average distance to other points.

Figure 3 summarizes all the steps of the RepSeg for a chromosome, including consecutive segment splitting (e.g., size 500 Kbp), FCGR generation (e.g., using $k=9$), distance matrix calculation, and representative segment selection based on the minimum average distance in the distance matrix. Figure 3 also includes a Multidimensional Scaling (MDS) representation of the distance matrix $D$, where matrix elements that are close to each other are depicted by closer dots in the MDS plot. The representative segment, highlighted in red, is the center of mass of the points in the MDS representation, as it has the minimum distance to all other points.

The proposed pipeline identifies the segment with the minimum average distance from all other segments of the genome, making it a strong candidate to serve as the representative segment, as it shares the most similarities with the other segments. However, this pipeline has three intrinsic limitations. First, it is sensitive to both the segment length and the $k$ value used in the $k$-mer generation for FCGR (using a different segment length and different $k$ can lead to different representative). Second, since the genome is divided into non-overlapping segments, the representative can only be selected from these segments, which means that it is not chosen from among all possible segments of the same length from the genome. Although this issue could be addressed by using overlapping segments, this approach would significantly increase the total number of segments, leading to higher computational and memory costs due to the quadratic time and space complexity of the distance matrix $D$. Finally, computing the distance matrix $D$ can be time-consuming for large genomes with a high number of segments, as it requires calculating the distance between all pairs of segments. To address these limitations, the segment length and $k$-value are consistent in our experiments to ensure comparability of results. Subsequently, an approximate pipeline for selecting the representative segment is proposed, which is capable of identifying a representative from any position across the genome, while simultaneously addressing the computational complexity of RepSeg.

### Approximate representative segment selection pipeline (aRepSeg)

The RepSeg can be computationally expensive due to the quadratic time complexity of distance matrix calculation, which makes its performance highly dependent on the genome length and the chosen segment length. To address this limitation, an alternative pipeline referred to as Approximate Representative Segment Selection Pipeline (aRepSeg) is proposed, which produces a representative segment that approximates the one chosen by RepSeg but with reduced time complexity. For this purpose, instead of dividing the genome into consecutive, non-overlapping segments, aRepSeg randomly selects $n$ fixed length segments and computes their pairwise FCGR distances. The number of these random segments affects the time complexity of the algorithm; increasing $n$ improves the approximation but also increases the computational cost. The genomic signature, which is pervasive within a species, ensures that the distances between segments of a genome generally exhibit low variability and remain relatively small. However, segments from regions with large tandem repeats can deviate significantly from this pattern, potentially skewing the results. To address this, aRepSeg incorporates a step to identify and exclude these outliers from the randomly selected segments.

More precisely, aRepSeg is an iterative pipeline that starts by initializing the dynamic set $\hat{S}$ with $n$ randomly chosen segments of fixed length. It then creates the distance matrix $\hat{D}$ by calculating the pairwise distances between the FCGR images of all segments in the set $\hat{S}$. After the calculation of the distance matrix, aRepSeg applies an outlier detection algorithm based on the Interquartile Range (IQR) to identify outlier segments within $\hat{S}$. Accordingly, the pipeline removes these outliers from the dynamic set and adds new randomly selected segments, equal in number to the removed ones. The pipeline iteratively recalculates the distance matrix, detects the outliers, and updates the $\hat{S}$ by replacing the outliers until no further outliers exist in the set. Finally, the segment with the minimum average distance to all others in $\hat{S}$ is selected as the representative.

To remove outlier segments from $\hat{S}$, an algorithm similar to the Interquartile Range (IQR) method is used, which is typically applied to identify and remove outliers. The IQR method works by calculating the interquartile range, which is the difference between the first quartile (Q1) and the third quartile (Q3) of the data^54^. The algorithm then defines a range for typical data by adding 1.5 times the IQR to Q3, known as the upper bound, and subtracting 1.5 times the IQR from Q1, known as the lower bound. Data points outside this range are considered outliers and are removed, resulting in a cleaner dataset with fewer extreme values. In the pipeline, the IQR method is applied to the average distance of each segment in $\hat{S}$ from the other segments, which is derived from the distance matrix $\hat{D}$. However, aRepSeg modifies this algorithm to only remove values above the upper bound, as small distances are expected, and only high distances pose a problem (they could result from, e.g., one of the segments originating from a tandem repeat region). Any segment detected as an outlier by this modified IQR-based method is removed from the set and replaced with a new random segment chosen from the genome.

To evaluate the effectiveness of aRepSeg and its ability to approximate RepSeg, the representative segments identified by each algorithm are compared. Rather than directly computing the distance between these two representative segments, which would not necessarily be informative, both RepSeg and aRepSeg first identify representative segments for a specific genome. Then, the genome is divided into non-overlapping, continuous segments, each of the same size as the representative segment. Distances are computed between each of these segments and the representative segments selected by RepSeg and aRepSeg, respectively. The Mean Absolute Error (MAE) between the two resulting distance vectors serves as a measure of the approximation error introduced by aRepSeg.

### *CGR-Diff*: a software for CGR comparison

To facilitate the experiments on the pervasiveness and variation of genomic signatures, *CGR-Diff*, a novel graphical software tool is developed to extract, visualize, and compare CGRs or FCGRs of different genomic sequences, including chromosomes, genome segments, or any other arbitrary DNA sequence stored in a FASTA file. The tool offers adjustable segment lengths, customizable $k$-mer sizes, and a selection of distance measures for comparison. The software includes a feature allowing users to utilize the assemblies suggested in Fig. 1b or upload their own assemblies for analysis. Additionally, users have the option to select chromosome segments from a list of annotated sequence segments, such as cytobands, if these annotations exist for the selected assembly (cytoband annotations are currently available only for the human genome). The tool also offers the ability to apply the reverse complement to a selected DNA sequence before generating the CGR/FCGR representation. See GitHub for more information regarding how to use the software.

Furthermore, the software includes built-in experiments designed to analyze genomic signature variations both within a species (intragenomic variation) and across different species (intergenomic variation). It also facilitates the replication of the RepSeg and aRepSeg analyses for entire genomes or individual chromosomes from various species. The details of these built-in analyses are as follows:

1. *Comparison of consecutive non-overlapping segments for each chromosome or entire genome*: In this experiment, the tool allows users to upload a FASTA file or select the entire genome or specific chromosomes of a species from a predefined list. Users can also specify a segment size and choose a distance measure. The sequence is then divided into consecutive non-overlapping segments of the chosen size. The software then calculates and displays the pairwise distances between neighboring segments, providing a visual representation of these distances on a plot. Additionally, it illustrates the FCGR of each DNA segment, quantifies the distance measures, and visualizes the differences between pairs of FCGRs.
2. *Comparison of different segments of a chromosome or genome with a reference sequence*: This experiment is similar to the first, except that the consecutive non-overlapping segments of a chromosome or genome are compared with a chosen, common reference sequence, instead of being compared with their neighboring segments. For the reference sequence, users can choose any segment from the same or different genome with arbitrary start and end points, or select a predefined segment from the list of annotated cytoband sections. The results of these comparisons are visualized in a plot, demonstrating the difference between the chosen reference sequence and each segment. To provide more detailed results, users can select any segment and visually compare its FCGR with that of the reference sequence. The software displays the FCGR of the selected segment, the FCGR of the reference sequence, and the distance between the two, enabling both qualitative and quantitative comparisons.
3. *Representative segment selection*: This experiment enables users to run representative selection pipelines on a desired sequence. Users can either upload a FASTA file or select a predefined chromosome or genome from a list. Afterward, they can specify the size of the representative segment, choose the selection pipeline (from RepSeg, aRepSeg, or Random Selection), and select a preferred distance measure. Upon execution, the software identifies and describes the representative segment (including its location in the sequence and its cytoband) and generates a plot showing the distances between different segments along the chromosome or genome and the representative segment. Additionally, it displays the FCGR of the representative segment and each of the segments at each step, along with their differences and distance values.

### Experimental design

This section provides a detailed account of the experiments conducted in this study, including the hyperparameter values used, such as segment length and the $k$ value for FCGR generation. To determine the optimal $k$-mer size and fragment length, both empirical and quantitative analyses were performed. For the empirical analysis, the FCGRs of human chromosome 1, as well as its first 500 Kbp, and its first 200 Kbp of the p34.1 cytoband (a euchromatin and gene-rich region), were visualized across $k$ values ranging from 2 to 9 (see Supplementary Fig. S1). This analysis showed that there is a trade-off between choices: FCGRs with smaller $k$ values have insufficient resolution, while FCGRs with larger $k$ values have sufficient resolution but can be faint and less discernible when the originating sequences are shorter. For the quantitative analysis, one-nearest-neighbor (1-NN) species classification tasks were performed while systematically varying both the $k$ value and the length of the segments used (see Supplementary Material Section A.3). This analysis showed that $k=4$, $k=5$, and $k=6$ yielded comparable classification accuracy. Overall, $k = 6$ achieved an optimal balance between visual resolution and classification performance, and was selected for most experiments where FCGRs were generated from chromosome segments rather than entire chromosomes. The exception was Exp 1, where the sequences were larger (full chromosomes) and a $k$ value of 9 was more effective, producing clear FCGR images at a resolution of $512 \times 512$ (see Supplementary Fig. S1). Regarding sequence length, both 200 Kbp and 500 Kbp segments achieved comparable classification performance, and these lengths were used interchangeably in different experiments depending on the genome length.

1. * Pervasive Nature of Genomic Signatures:* This experiment empirically investigates the pervasive nature of genomic signatures across the genome by visualizing the FCGRs of the full sequences of all 24 human chromosomes and all 10 maize chromosomes using $k$-mers with $k=9$. This choice of $k$ produces high-resolution FCGR images, which are empirically verified for representing the genomic signature of the full length of chromosomes. The resolution is particularly critical for detailed visualization, as even the shortest chromosome in these species, human chromosome 21, is approximately 45 Mbp.
2. *Distance Selection:* The majority of the experiments are built upon comparing the signature of two DNA sequences using their FCGR images. However, comparing two FCGRs using different distance measures can sometimes lead to non-comparable distance values, as each of them employs a unique method of comparison. Moreover, in FCGR images, it is not only the distribution of $k$-mers that matters; the geometry and visual patterns embedded in the images also contain valuable information that is pervasive and species-specific^20^. Therefore, it is important to analyze the behavior of different distance measures against biological expectations, when comparing FCGR images. For example, if two DNA sequences are phylogenetically similar, the distance value between their FCGRs is expected to be small; otherwise, a larger distance value is expected. To evaluate the effectiveness of the eight distance measures introduced in the section *Methods: Distance Measures* and to determine the optimal one for comparing FCGRs, two experiments are conducted: *Human Intragenomic Distance Analysis*, and *Intergenomic Distance Analysis*.
  In the *Human Intragenomic Distance Analysis* experiment, the sequence length varies depending on the region of interest. However, when selecting a random segment from a region, a standard length of 500 Kbp is used. This segment size provides a proper sample size that captures sufficient variation. Since each chromosome spans several Mbp, selecting multiple random 500 Kbp segments ensures diverse sampling and meaningful comparisons. Furthermore, the probability of significant overlap between two randomly selected 500 Kbp segments is very low. For instance, the shortest tandem repeat region within the human chromosome is 10 Mbp, belonging to chromosome 14. Within this region, the chance of observing more than 70% overlap between two randomly selected segments of 500 Kbp is approximately 3%, while the probability of exceeding 50% overlap is only about 5%. These low probabilities ensure that the selected segments are largely independent when the segment size is 500 Kbp, while larger segments would increase the risk of overlap, and smaller segments may fail to capture the genomic signature adequately. Also, the results of the one-nearest-neighbor species classification tasks (see Supplementary Material Section A.3 for experimental details and Table S1 for results), in which both $k$-mer size and segment length were systematically varied, indicated that 500 Kbp segments generally yield higher accuracy than shorter segment lengths. Accordingly, we use the same 500 Kbp segment length in the *Intergenomic Distance Analysis* to ensure consistency.
3. (*Human Intragenomic Distance Analysis*) focuses on comparing FCGRs within the human genome, leveraging the complexity of this genome and the extensive annotations available for its chromosomes, such as the color-coded regions in the NCBI Data Viewer. In this study, a series of experiments is conducted using various distance measures to compare FCGRs across different human chromosomal regions, including short and long tandem repeats, as well as regions with varying G+C content and CpG composition, such as telomeres, heterochromatin, euchromatin, and the p-arms of acrocentric chromosomes. The effectiveness of these distance measures is evaluated based on their consistency with known differences in sequence composition, such as the occurrence of regional clustering of short and long tandem repeat sequences. This comprehensive intragenomic analysis on the human genome enables the assessment of how well different distance measures align with biological expectations regarding the occurrence and length of tandem repeating sequences. The series of experiments conducted in this study is briefly described below, with detailed explanations provided in the Supplementary Material Section A.2.

- *Telomere vs. Telomere* aims to determine the distances between the p-arm telomere of chromosome 1 and the p-arm telomeres of other chromosomes. Telomeres, composed of conserved tandem repeats and associated proteins, exhibit similar structure and composition across human chromosomes^55,56^.
- *Heterochromatin vs. Heterochromatin* calculates the average distance between the most condensed heterochromatic region (located distal to the centromere on the p-arm, or, if absent, proximal on the q-arm) and other heterochromatic regions within the same chromosome. The final result is the overall average distance calculated across all chromosomes. Heterochromatin, characterized by high condensation and transcriptional inactivity^57,58^, is visually represented by black and three shades of gray in the NCBI Data Viewer, with darker shades indicating higher levels of compaction^59^.
- *Heterochromatin vs. Euchromatin* calculates the average distance between the most condensed heterochromatic region (located distal to the centromere on the p-arm or proximal on the q-arm if absent) and four randomly selected euchromatic segments within each chromosome. The final result is the overall average distance computed across all chromosomes. Euchromatin, characterized by less condensed DNA and active gene transcription^60^, differs significantly from heterochromatin in structure, function, and genomic composition^60,61^.
- *p-arm vs. q-arm* measures the average distance between 100 randomly selected 500 Kbp segments on the p-arm and q-arm of each acrocentric chromosome (13, 14, 15, 21, 22) as well as the Y chromosome. Acrocentric chromosomes are characterized by a short p-arm, which is enriched with tandem repeat sequences, and a longer q-arm, which contains fewer repeats^62,63^. These tandem repeat regions consist of long DNA stretches where a sequence is repeated in a head-to-tail manner, varying in repeat unit length, size, and organization^64–66^. Since the Y chromosome contains repetitive regions in its q-arm^3,67^, it is also included in this experiment.
- *Y q-arm vs. acrocentric chromosome p-arm (Large Tandem Repeat Arrays)* calculates the distance between the tandem repeat arrays on the q-arm of the Y chromosome and those on each acrocentric chromosome, and reports the average. The q-arm of the Y chromosome contains a repetitive region that differs in length and in the nature of its tandem repeat arrays from those on acrocentric chromosomes^3^.
- *Arbitrary Sequences* calculates the average intragenomic distance by selecting two random non-overlapping 500 Kbp sequences from a randomly chosen chromosome, computing the distance between their FCGRs, and averaging the results over 100 iterations. Among the discussed experiments, the *Telomere vs. Telomere* comparison is expected to yield the smallest distance due to the identical repeats in the telomere regions. The *Heterochromatin vs. Heterochromatin* comparison should produce the second smallest distance, and it should be smaller than the *Heterochromatin vs. Euchromatin* comparison. The *p-arm vs. q-arm* experiment, which compares repeat-rich regions to non-repetitive sequences, is expected to result in the largest distance among all tests. The *Large Tandem Repeat Arrays* experiment is expected to show a large distance, but smaller than *p-arm vs. q-arm* since it compares different types of repeats and explores the variation within the repeats, specifically between Y q-arm repeats and acrocentric p-arm repeats. Finally, the *Arbitrary Sequences* test is anticipated to reflect an intermediate intragenomic distance within the human genome, as its large sample size ensures a balanced sampling of diverse combinations of genomic sequences.
  **Exp 2.2** (*Intergenomic Distance Analysis*) involves comparing random segments from the human genome with random segments from eight other species listed in Subset 2 of Fig. 1b. Since FCGR images are species-specific, it is hypothesized that phylogenetic distances between the genomes of different species and the human genome will be reflected in their FCGR comparisons, with distances between two random human genome sequences expected to be smaller than those between human and non-human genome sequences. Smaller distances are also anticipated between human and chimpanzee genome sequences compared to those involving human genome and genomes from species in different kingdoms. This evaluation enables the assessment of each distance measure based on how well it aligns with known phylogenetic or evolutionary distances.
  To perform this analysis, 100 random segments of length 500 Kbp are selected from the human genome to serve as reference sequences, ensuring that centromeres and large tandem repeat arrays (e.g., the short arms of acrocentric chromosomes and the long arm of the Y chromosome) are excluded. Selections from telomeric regions are not excluded due to their short sequence footprints and minimal impact on the *k*-mer compositions of FCGRs. Then, for each of the nine species (human and eight others), 100 random segments of the same length (500 Kbp) are selected, and pairwise distances between their FCGRs and the reference FCGRs from the human genome are calculated using all eight distance measures. When selecting random segments from the human genome, centromeres and large tandem repeat regions are again avoided. Finally, these distances are compared across all nine species using boxplots and the Wilcoxon signed-rank test^68,69^, with expectations based on known phylogenetic relationships. The Wilcoxon signed-rank test is applied because the same reference human FCGRs are used to compute both sets of distances, leading to dependent matched samples.
  Exp 3 *Intragenomic Variation*: A series of experiments is conducted to evaluate the RepSeg and aRepSeg methods. The methods that are designed to select representative genomic segments for various species. These representatives serve multiple purposes, including exploring variation in genomic signatures along chromosomes or entire genomes and supporting downstream tasks, such as the species classification in Exp 4. Experiments in this section, focus on the human and three species listed in Subset 3 of Fig. 1b. For human and maize, which have long genomes (approximately 3 billion bp and 2 billion bp, respectively) and chromosomes ranging from 45 Mbp (e.g., human chromosome 21) to 300 Mbp (e.g., maize chromosome 1), a representative segment is selected for individual chromosomes to maintain their distinct genomic information. For species with smaller genomes, such as *Aspergillus nidulans* and *Dictyostelium discoideum*, all chromosomes are concatenated with ‘N’ to prevent unwanted $k$-mers, and a representative segment is selected for the entire genome. In all cases, a segment length of 500 Kbp is used, as it is sufficiently long to preserve genomic signatures while remaining a small fraction of the total genome length (e.g., less than 1% of the shortest human chromosome). Furthermore, for comparing FCGRs in these experiments, the DSSIM distance measure is utilized, as it demonstrates the best compatibility with the biological expectations discussed in the Distance Analysis experiment (see *Results* section).
  **Exp 3.1** (*Representative Segment Selection*) applies the RepSeg to the human genome and the genome of each species listed in Subset 3 of Fig. 1b. This pipeline finds a representative segment for each individual chromosome in human and maize, and for the entire genome in *Aspergillus nidulans* and *Dictyostelium discoideum*. Using the representative segment as a reference, the experiment calculates and plots the distances between each consecutive segment (of the same length as the representative segment, i.e., 500 Kbp) and the representative segment. This analysis highlights the prevalence or variation of the genomic signature within a single chromosome or the entire genome.
  **Exp 3.2** (*Approximate Representative Segment Selection*) repeats the previous experiment on the human genome and the genomes of all the species of Subset 3 in Fig. 1b, but uses the aRepSeg to determine the representative segment. The aRepSeg utilizes a set of random segments $\hat{S}$, and in this experiment, the size of $\hat{S}$ is set to 30. This size is determined through an empirical experiment (see Supplementary Table S5)..

**Exp 3.3** (*Unlikely Representative Segment from Tandem Repeat Regions*) is an experiment similar to the previous ones but designed to demonstrate the impact of selecting a random segment from regions with large and small tandem repeats, such as the centromere, as the representative segment. This experiment is applied only to human chromosomes, as it is the only genome from Fig. 1b that contains annotations for cytobands and repeat regions.

Exp 4 *Taxonomic Classification*: To evaluate the utility of the representative segment chosen by RepSeg or aRepSeg in downstream tasks, a simple taxonomic classification experiment is conducted using a one-nearest-neighbor (1-NN) classifier. This experiment is applied to all the species listed in Fig. 1b, except for *Paramecium caudatum*, whose genome is available only at the scaffold level, making it unsuitable for consistent segmentation or classification. For this experiment, the segment size is reduced from 500 Kbp, as used in previous experiments, to 200 Kbp. This adjustment accommodates the relatively short genomes of some species in the dataset, where extracting 100 random 500 Kbp segments per species would be impractical. This choice is further supported by the analysis in Supplementary Material Section A.3, which shows that 200 Kbp provides reliable species classification accuracy.

**Train Data**: The training dataset consists of one representative for each species, which is selected under two scenarios: (1) using the pipelines (RepSeg or aRepSeg) or (2) choosing a random segment from a chromosome or genome as the representative. For genomes shorter than 100 Mbp, in the first scenario, the representative is selected by applying RepSeg or aRepSeg to the entire genome, whereas in the second scenario, the genome is divided into consecutive segments of 200 Kbp, and one segment is randomly chosen as the representative. For genomes longer than 100 Mbp, the representative in the first scenario is determined as the final representative of the representatives of individual chromosomes, where the final representative is the segment with the minimum average distance to all other chromosome-level representatives. In the second scenario, a random chromosome is first selected, divided into consecutive segments of 200 Kbp, and a random segment from these is chosen as the representative.

**Test Data**: The test dataset consists of 100 random segments of 200 Kbp each, extracted from the entire genome for species with genomes under 100 Mbp and from random chromosomes for species with genomes exceeding 100 Mbp. This results in a total of 1,100 test samples, representing 11 species.

**Classification Approach**: The classifier predicts the label for each test sample by comparing the DSSIM distance between the FCGR of the test sample and the FCGRs of the 11 representative segments in the training set. The DSSIM distance measure is used for comparing FCGRs, as described in Exp 3.

**Evaluation**: The two scenarios are compared by computing their classification accuracy. To avoid bias towards tandem repeat regions, for the random selection scenario, the average accuracy over 50 repetitions is reported. This comparison assesses the effectiveness of the pipelines (RepSeg or aRepSeg) in generating representative segments for downstream tasks.

## Results

### *CGR-Diff* software tool for assessing intragenomic variations in the genomic signature

**Fig. 4:**
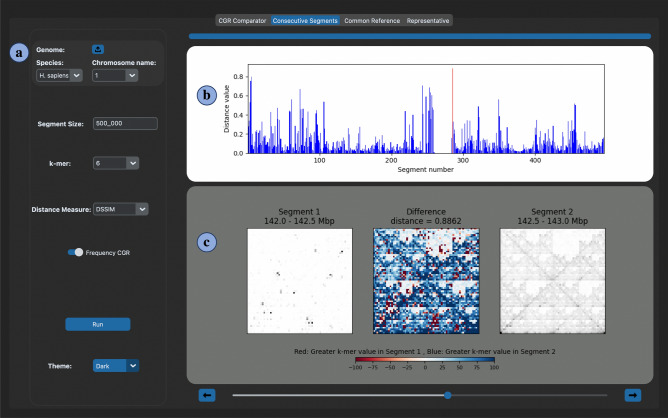
**A screenshot of the *consecutive non-overlapping segments *experiment of the CGR-Diff software.** (**a**) Control panel showing the parameters of the experiment, including $k$, segment size, and distance measure. (**b**) Plot displaying the distances between FCGRs of consecutive segments across the first human chromosome (using $k=6$, segment size = 500 Kbp, and distance measure = DSSIM). The red bar indicates the maximum distance (0.88) at the boundary between a tandem repeat region (q12) and a euchromatic region (q21.1). (**c**) FCGRs correspond to two consecutive segments associated with the maximum distance, with their positions on the chromosome mentioned at the top of the images. The left and right images show the individual FCGRs, and the center shows their pixel-wise difference, highlighting shifts in $k$-mer composition.

Figure 4 demonstrates the capabilities of the software tool by presenting an example from its first built-in experiment, *Comparison of consecutive non-overlapping segments for each chromosome or entire genome*. The primary objective of this experiment is to evaluate intragenomic variation in the genomic signature and to quantitatively compare genomic signatures along the chromosome. In this example, human chromosome 1, the longest chromosome in the genome, is analyzed using a segment size of 500 Kbp, $k = 6$ for FCGR generation, and DSSIM (as defined in *Methods: Distance Measures*) as the distance measure to capture variation along the chromosome.

Figure 4b displays a feature of the software that visualizes the DSSIM distance between each pair of consecutive genomic segments. As seen in this figure, in the case of human chromosome 1, most of these distance values are low (with an average of approximately 0.11), indicating a consistent genomic signature between neighboring segments. However, several high distance values are observed to occur at specific locations, particularly at cytoband boundaries, reflecting significant changes in $k$-mer composition. The highest observed distance in this experiment is 0.88 (highlighted in red), which is a significant value given that DSSIM ranges from 0 to 1 when comparing FCGR images. This peak occurs at the boundary between the q12 and q21.1 cytobands of chromosome 1, where q12 is associated with tandem repeats, while q21.1 corresponds to a euchromatin region. Before this peak, there is a portion of the plot where the DSSIM distances are close to zero. This low variation occurs within the tandem repeat region (q12), where the sequences of consecutive segments are highly similar, resulting in similar FCGR patterns and low DSSIM distance values between them. 

Figure 4c displays a feature of the software that provides a detailed visualization of two selected consecutive segments and their comparison. In this example, the two selected consecutive segments are those whose distance is the peak distance value in Fig. 4b (segments 285 and 286, together spanning 142.0–143.0 Mbp). The left and right images display the FCGRs of the individual segments, while the center matrix shows the pixel-wise difference between them, highlighting which $k$-mers are more abundant in each segment. The observed difference in FCGR patterns reveals a shift in sequence composition that aligns with the transition from a tandem repeat-rich region (Segment 1) to a euchromatic region (Segment 2).

**Fig. 5:**
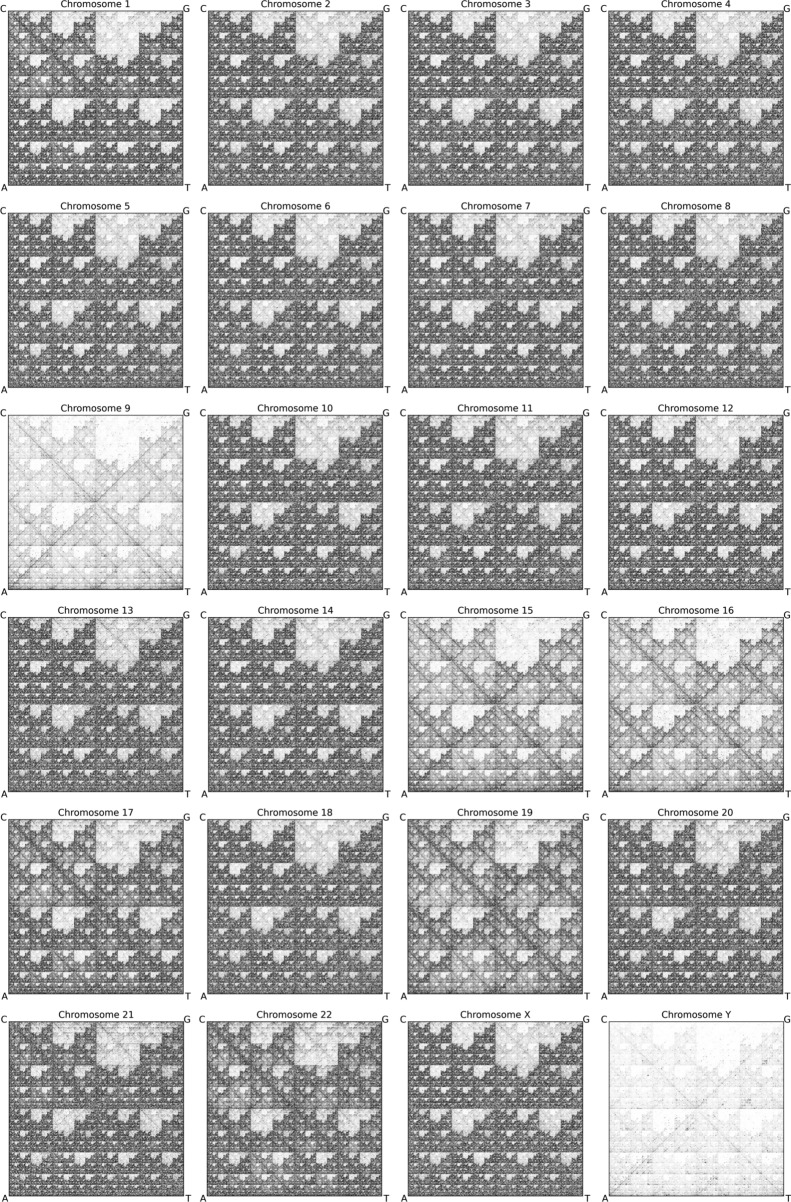
**Analyses of the human genome genetic signature.** Each image represents the FCGR of a complete human chromosome, constructed using $k = 9$. The overall structure reveals a preserved genomic signature across chromosomes, while variations in intensity indicate differences in $k$-mer distribution. Specifically, certain chromosomes, such as chromosome 9, 15, 16, and Y, appear lighter, consistent with the presence of regions with known high $k$-mer repetition.

### Demonstrating the pervasive nature of genomic signatures (Exp 1)

The FCGRs for the complete sequences of all 24 human chromosomes (see Fig. 5) and all 10 maize chromosomes (see Supplementary Fig. S2) are generated using $k = 9$. The FCGRs of human chromosomes exhibit similar geometric patterns across all chromosomes, which are distinctly different from those observed in maize. Similarly, the FCGRs of maize chromosomes are consistent among themselves. These observations support the assumption that genomic signatures are both species-specific and pervasive across the entire genome of a species.

Despite the overall consistency of FCGR patterns across all human chromosomes, chromosomes 9, 15, 16, and Y exhibit notable deviations in FCGR intensity, as shown in Fig. 5. These variations are not due to chromosome length, but rather to the high relative frequencies of some specific $k$-mers (9-mers in Fig. 5), which result in lower counts of the other $k$-mers (lighter other regions in the FCGR image). Specifically, the relative frequencies of AAGGTAAGG (chromosome 9, 0.75%), CTTACCTTA (chromosome 15, 0.34%), TTACCTTAG (chromosome 16, 0.36%), and TAAGGTAAG (chromosome Y, 1.00%) are significantly higher than the relative frequency of 9-mers in the other chromosomes (average 0.13%). These observed high frequencies of specific 9-mers are likely the result of extensive tandem-repeat regions and segmental duplications within chromosomes 9, 15, 16 and Y, that are absent in the other chromosomes (see Supplementary Table S2). For example, chromosome 9 contains the largest block of heterochromatin among human chromosomes and exhibits numerous repetitive regions within the centromere and large heterochromatic regions^70^. Similarly, chromosomes 15 and 16 have some of the highest levels of segmental duplications in the human genome^71,72^. Finally, the human Y chromosome is substantially different from all other chromosomes, as it is densely packed with repeats, such that almost any sequence from the Y chromosome either repeats internally or has a near-identical copy elsewhere on this chromosome^3,73^.

A similar phenomenon is observed in the maize chromosomes, with the FCGR images of chromosomes 2 and 4 displaying reduced overall pixel intensities. These lighter FCGRs are due to the high relative frequencies of specific $k$-mers (see Supplementary Table S3). Specifically, the relative frequencies of TCATCATCA (chromosome 2, 0.10%) and TGATGATGA (chromosome 4, 0.05%), are higher than the relative frequency of 9-mers in the other chromosomes (average 0.02%).

### Determining the optimal distance measure (Exp 2)

#### Human intragenomic comparison (Exp 2.1)

**Fig. 6:**
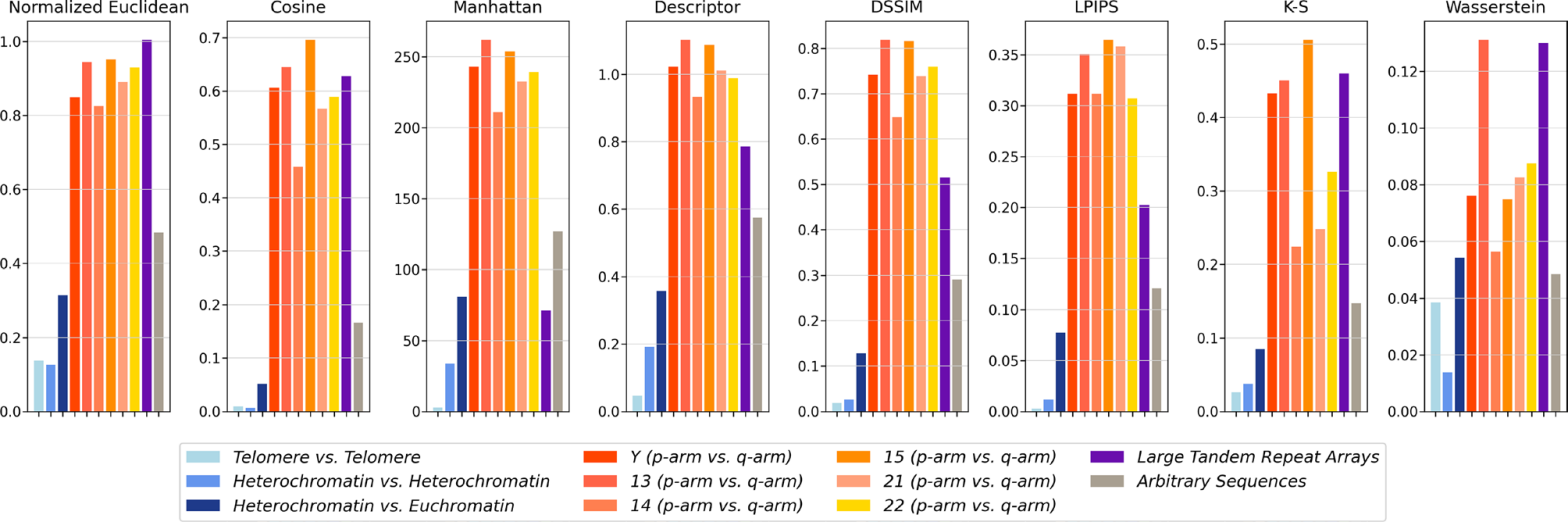
**Intragenomic Distance Analysis (Exp 2.1).** Each bar plot shows the performance of a distance measure across the different experiments in Exp 2.1. Three shades of blue represent the *Telomere vs. Telomere*, *Heterochromatin vs. Heterochromatin*, and *Heterochromatin vs. Euchromatin* experiments, which are expected to yield relatively small distance values compared to the other experiments (the lighter the shade, the smaller the expected distance value). Six warm colors correspond to the *p-arm vs. q-arm* experiment in different chromosomes (Y, 13, 14, 15, 21, and 22), where distance values are expected to be the largest among all experiments. Purple bars represent the *Large Tandem Repeat Arrays* experiment, which is expected to have large distance values, though smaller than those in the *p-arm vs. q-arm* experiment. Finally, gray bars indicate the *Arbitrary Sequences* experiment, which is expected to produce intermediate distance values. Among all distance measures, Descriptor, DSSIM, and LPIPS align best with biological expectations.

The performance of the eight distance measures in the human intragenomic distance analysis experiment is shown in Fig. 6. Considering the identical sequence repeats in telomeres, the *Telomere vs. Telomere* comparison is expected to yield the smallest distance relative to other experiments. As seen in the figure, this expectation is met for most distance measures, except for the Wasserstein distance (where the *Telomere vs. Telomere* comparison is considerably larger than *Heterochromatin vs. Heterochromatin*) and for the Normalized Euclidean and Cosine distances (where *Telomere vs. Telomere* is slightly larger than *Heterochromatin vs. Heterochromatin*, but still comparable). Additionally, the expectation that the *Heterochromatin vs. Euchromatin* comparison would result in a larger distance than the *Heterochromatin vs. Heterochromatin* comparison is confirmed by all distance measures. The *p-arm vs. q-arm* experiment in different chromosomes is expected to produce the largest distance among all tests, as it compares repeat-rich regions to non-repetitive sequences. However, as shown in Fig. 6, the Normalized Euclidean, Cosine, K-S, and Wasserstein distance measures fail to reflect this expected result. Finally, in the *Large Tandem Repeat Arrays* experiment, a large distance is expected, greater than in the *Arbitrary Sequences* comparison but smaller than in the *p-arm vs. q-arm* comparison, since it compares different types of large tandem repeat arrays. However, the Normalized Euclidean, Cosine, Manhattan, K-S, and Wasserstein distance measures fail to meet this expectation. Overall, among all the distance measures, Descriptor, DSSIM, and LPIPS performed as expected in Exp 2.1.

#### Intergenomic comparisons (Exp 2.2)

**Fig. 7:**
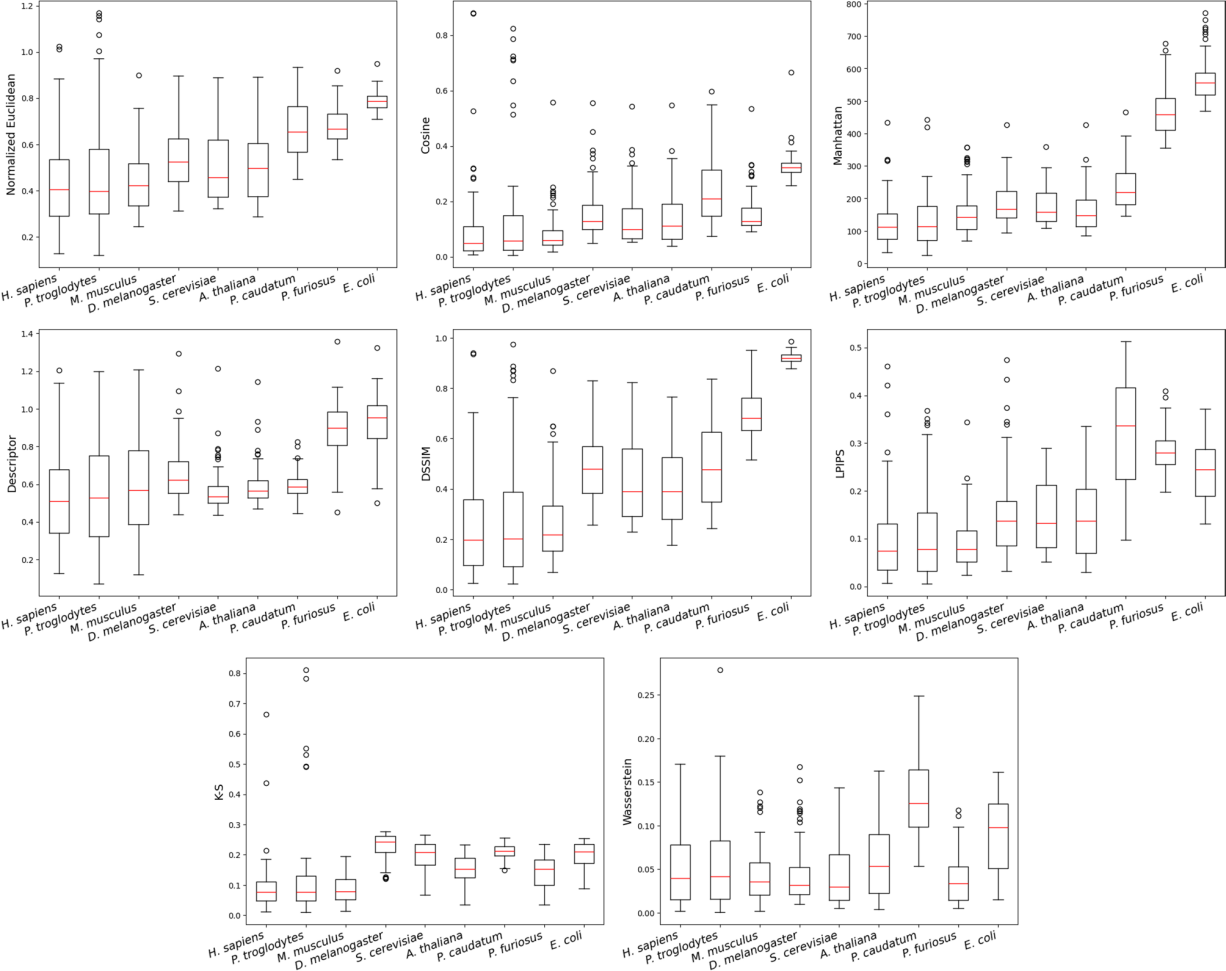
**Intergenomic Distance Analysis (Exp 2.2).** The eight panels correspond to the eight different distance measures considered. Each panel presents boxplots illustrating the distribution of pairwise distances between 100 randomly selected human reference segments and 100 randomly selected segments from the same, or one of eight other species (shown on the horizontal axis). In each boxplot, the box represents the interquartile range, the whiskers indicate the broader spread of values, and the red line marks the median.

Figure 7 presents boxplots illustrating the distribution of intergenomic distance values across species with known phylogenetic distances from the human genome, using the eight proposed distance measures. Each boxplot represents the distribution of pairwise distances between 100 randomly selected human segments (referred to as human reference segments) and 100 randomly selected segments from the corresponding species. In these boxplots, the box corresponds to the interquartile range, the whiskers indicate the broader spread of values, and the red line marks the median. In the arrangement of the boxplots, the first plot, positioned on the far left, corresponds to comparisons between human reference segments and randomly selected human genome segments (human-human). Progressing to the right, the subsequent boxplots represent increasing phylogenetic distances, comparing human reference segments to segments from increasingly distantly related species. This arrangement illustrates the expected trend of increasing intergenomic distances as we move from closely related species (e.g., *Pan troglodytes* (chimpanzee) and *Mus musculus* (mouse)) to those less related, and further to species from different kingdoms.

Based on biological hypotheses, an increase in distance values is expected from left to right along the horizontal axis in each plot, reflecting greater divergence from the human genome. Smaller distances are anticipated for comparisons between human reference segments and randomly selected human genome segments (human-human) compared to distances between human reference segments and segments from other species. Furthermore, no significant difference (i.e., $p$-value greater than 0.05) is expected between the human-human distances and the distances between human reference segments and segments from closely related mammals, such as *Pan troglodytes* (chimpanzee) and *Mus musculus* (mouse). In contrast, significant differences are expected when comparing the human-human distances to the distances between human reference segments and segments from species in different kingdoms.

Moreover, the variability in distance distributions when comparing each species to human reference segments reflects the intragenomic variation within that species. This variation is due to known intragenomic sequence composition, particularly in relation to large tandem repeat regions. Therefore, the variability of each boxplot can also be assessed based on biological predictions. For instance, the genome composition of vertebrates leads to an expectation of greater variability compared to bacterial genomes. Notably, the chimpanzee genome is predicted to exhibit a high variation, highlighting its abundant intragenomic differences. 

According to Fig. 7 and the $p$-values from the Wilcoxon signed-rank test in Supplementary Table S4, no statistically significant differences are observed between the human-human distances and the human-*P. troglodytes* (chimpanzee) distances across the eight distance measures. However, when comparing the human-human distance values with those of human-*M. musculus*, the Manhattan, Descriptor, and Wasserstein distance measures yield statistically significant differences, with $p$-values of 0.0009, 0.0348, and 0.0415, respectively. Therefore, these three distance measures, do not support the expectation of no significant difference among mammals. Additionally, when comparing the human-human distances with the distances between human and more distantly related species, all distance measures show statistically significant differences, except for Wasserstein distance in the comparison between human-human and human-*A. thaliana* ($p = 0.0684$). Among the remaining distance measures, Normalized Euclidean, Cosine, and DSSIM most accurately reflect phylogenetic distances and align with the expected variation in vertebrates.

#### Conclusion of Exp 2: DSSIM confirmed as preferred distance measure

Considering the results of Exp 2.1 and Exp 2.2, DSSIM is the preferred distance measure for comparing FCGRs and is used in subsequent experiments. The consistent performance of DSSIM is further supported by the Wilcoxon signed-rank test, where the *p*-value for the comparison between the human-human distances and those of human-*P. troglodytes* is 0.178, between human-human and human-*M. musculus* is 0.096, and for all other species, the $p$-value is less than $10^{-11}$. The DSSIM distance measure not only meets all biological expectations but also, compared to distance measures like LPIPS (as defined in *Methods: Distance Measures*), has lower computational complexity. Additionally, it has the advantage of boundedness, with a practical range of [0, 1], which makes the interpretation of numerical results easier.

### Evidence of low intragenomic variability of the genomic signature (Exp 3)

To quantitatively measure the intragenomic variation of genomic signatures within each chromosome of humans and maize, as well as across the entire genomes of *Aspergillus nidulans* and *Dictyostelium discoideum*, the RepSeg and aRepSeg are applied as described in Experiments 3.1 and 3.2. These pipelines extract representative segments, and the distances between consecutive segments (each 500 Kbp in length) and the representative segments are calculated.

**Fig. 8:**
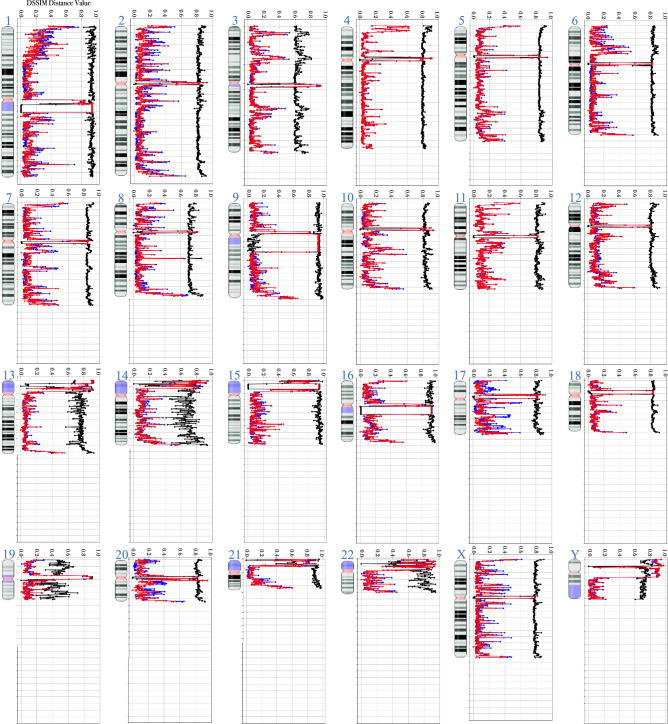
**DSSIM distances of consecutive segments from the representative segment across all human chromosomes.** Each chromosome is divided into successive non-overlapping 500 Kbp segments, and the distances are calculated from these to a chromosome representative segment. The red line (Exp 3.1) corresponds to a representative segment selected using the RepSeg method, where the representative is chosen as the segment with the smallest average distance to the others. The blue line (Exp 3.2) corresponds to a representative segment selected using the aRepSeg method, where the selection is approximated iteratively. The black line (Exp 3.3) corresponds to a representative segment randomly selected from a region with highly repetitive sequences. Each point along a line indicates the DSSIM distance between a 500 Kbp genomic segment and its representative segment. The horizontal axis shows the DSSIM distance values ranging from 0 to 1 (with increments of 0.2), where higher values indicate greater dissimilarity. The vertical axis is divided into intervals of 20, corresponding to sequential 500 Kbp segments along each chromosome. Chromosome ideograms to the left of each plot are adapted from the NCBI Genome Data Viewer^39^ and display key structural regions such as heterochromatin (regions colored in black and three shades of gray), euchromatin (white regions), centromeres (pink regions), and large tandem repeat arrays (purple regions).

Figure 8 presents the results of these experiments for human chromosomes. The line plots show the DSSIM distances of segments relative to their representative segments. In the red lines, the representative segment is selected using the RepSeg method, which follows a deterministic pipeline using all non-overlapping consecutive segments. The blue lines show the distances of the segments from the representative segment selected using the aRepSeg method, which employs an approximation pipeline based on a random sample of segments. Finally, the black lines illustrate the distances of consecutive segments from a randomly selected segment within regions of high repetitive sequences, as described in Exp 3.3. Figure 8 also includes ideograms of human chromosomes (adapted from the NCBI Genome Data Viewer^39^).

According to the red lines in Fig. 8, among all human chromosomes, the majority of segments (83.32%) exhibit a DSSIM distance of less than 0.24 from the representative segment suggested by RepSeg. As shown in Supplementary Fig. S6, a DSSIM distance of 0.24 can result from altering only about 1.1% of a 500 Kbp sequence, indicating that such a value can result from small structural composition changes. The similarity of FCGR patterns across most segments confirms the pervasiveness of genomic signatures within the chromosomes. This finding aligns with the results of Experiment 1, which provided evidence for the pervasiveness of the genomic signature across different chromosomes, further supporting their pervasiveness within individual chromosomes as well. Consequently, it is reasonable to propose a shorter representative segment for a chromosome with millions of base pairs that effectively captures this signature. At the same time, a small fraction of segments (16.67%) exhibit outlier behavior, with FCGR patterns that differ from the majority and contribute to the observed fluctuations in the genomic signature along the chromosome. Several factors may underlie this intragenomic variation and require further investigation. As a case study, we examined the effects of common interspersed repeats–SINEs, LINEs, LTRs, and Satellites–on genomic signature variation in chromosome 1 and in the human chromosomes with the highest variability, namely chromosomes 9, 15, 16, and Y (see Supplementary Material Section A.4, Supplementary Fig. S7 and Supplementary Table S6). Our analysis suggests that these interspersed repeats do not contribute substantially or globally to the intragenomic variability profile, as overall differences between masked and unmasked DSSIM mean values remain small. Instead, the observed variation is primarily driven by distinct sequence composition in regions such as the centromeres, as well as by long noncoding DNAs, which have reduced G and C content and GC skew. The exception is chromosome Y, where tandem repeats dominate the chromosome to such an extent that, unlike the other chromosomes, the representative segment is selected from the repeat-rich q-arm (consequently, in chromosome Y, the intragenomic variability arises mainly from segments located in the p-arm).

As the genomic signature is consistently repeated along the chromosome, one may assume that a randomly selected segment from the chromosome can also serve as the representative. However, selecting a purely random segment as the representative is not advisable, as a randomly chosen segment from the outlier segments is an unlikely representative and would fail to accurately encapsulate the genomic signature (see the substantial difference between the black and red lines in Fig. 8 with an average MAE of 0.61 across all chromosomes). On the other hand, the close resemblance (average MAE of 0.02 across all chromosomes) between the blue line (aRepSeg) and red line (RepSeg) further validates the effectiveness of aRepSeg in accurately capturing the genomic signature while reducing the computational cost of RepSeg.

Supplementary Table S5 shows the effect of the hyperparameter $n$ (the size of the set $\hat{S}$ in aRepSeg) on runtime improvement and MAE between aRepSeg and RepSeg. In this table, an $n$ value of 1 corresponds to random representative selection. Intuitively, *n* determines the size of a dynamic set from which the representative segment is selected–only if the set contains no outliers. A larger $n$ increases the chances of including a high-quality representative segment; however, larger values of $n$ also increase time complexity, thereby reducing the time-saving advantage of aRepSeg. Therefore, identifying an optimal $n$ is essential to balance both time efficiency and effective representative selection. Computational analysis, which explored values of between 1 and 50, empirically determined that $n=30$ achieves an MAE of 0.027 on human chromosome 1, which is a negligible error within the range of DSSIM distances and corresponds to approximately a 20x improvement in computational time for representative selection in this chromosome. Overall, setting the aRepSeg hyperparameter $n$ to the optimal value of 30 achieves a good balance between precision and computational efficiency.

In summary, the study confirms that a representative segment of 500 Kbp, as suggested by both RepSeg and aRepSeg, effectively captures the genomic signature within chromosomes. This representative facilitates intragenomic variation analysis, and quantitative exploration of the pervasiveness of genomic signatures along individual chromosomes of the human genome, while highlighting outlier regions that show abnormal genomic signatures. If the goal is to select a human-genome-representative, the RepSeg pipeline would be augmented by a selection of the chromosome-representative with the smallest mean distance to the others (from the set of chromosomes 1-22, X, and Y chromosome-representatives).

To demonstrate the generalizability of RepSeg and aRepSeg beyond the human genome, these pipelines are applied to ten maize chromosomes (see Supplementary Fig. S3) as well as the entire genomes of *Aspergillus nidulans* and *Dictyostelium discoideum* (see Supplementary Fig. S4). The results from these species are consistent with those observed in Fig. 8, further supporting the pervasive nature of genomic signatures across both chromosomes and entire genomes. Some variations are observed, primarily due to the presence of tandem repeat regions, which is a pattern also seen in the human genome.

Across all maize chromosomes, the majority of segments (93.23%) exhibit a DSSIM distance of less than 0.24 from the representative segment selected by RepSeg. Furthermore, aRepSeg closely replicates RepSeg across all chromosomes, with an average MAE of 0.02. Also, similar to the human genome, DSSIM distances in the maize genome tend to increase in segments associated with centromeres and large tandem repeat regions.

The assembly of *Aspergillus nidulans* consists of eight chromosomes, which are concatenated to form the full genome sequence. Since the individual chromosomes have an average length of 3.7 Mbp, the 500 Kbp representative selection pipeline is applied to the concatenated chromosomes rather than to individual ones. Similarly, *Dictyostelium discoideum* has six chromosomes with an average length of 5.6 Mbp, and the same 500 Kbp representative segment selection pipeline is applied to the whole genome obtained by concatenation of all chromosomes. Supplementary Fig. S4 illustrates the DSSIM distance of consecutive segments from the representative segment selected by RepSeg (red line) and aRepSeg (blue line) for each species. Compared to the human and maize genomes, these two eukaryotes exhibit a more uniform distance from the representative segment, further supporting the pervasiveness of genomic signatures within the genome of a species. In *Aspergillus nidulans*, the average DSSIM distance from the representative segment selected by RepSeg is 0.13, with a standard deviation of 0.03, indicating low intragenomic variation. Also, aRepSeg closely aligns with RepSeg, with an MAE of 0.01. A similar trend is observed in *Dictyostelium discoideum*, where the average DSSIM distance is 0.004 (standard deviation: 0.006), and aRepSeg deviates from RepSeg with an MAE of 0.0003.

### Effectiveness of genomic signatures for alignment-free taxonomic classification (Exp 4)

To demonstrate the effectiveness of the representative segment selection pipelines, a simple taxonomic classification task is performed using 1,100 randomly selected segments from the species listed in Fig. 1b. First, a training set is constructed by selecting the representative segment identified by either RepSeg or aRepSeg for each species. Then, test samples are classified based on their DSSIM distance to the corresponding representative segment, achieving an accuracy of 84.91% when using RepSeg and 84.45% when using aRepSeg to select the representative segments.

Furthermore, to assess the significance of the representative segments selected by the pipelines, the classification task is repeated using a randomly chosen segment from each species as the reference instead of the representative segment suggested by the pipelines. This substitution results in a drop in average classification accuracy to 77.63% over 50 runs. This reduction in accuracy highlights the effectiveness of representative segment selection for downstream applications such as taxonomic classification. Additionally, it suggests that while genomic signatures are pervasive across the genome of a species, they are not entirely uniform, likely due to the presence of repetitive regions.

Most misclassifications occur between human and chimpanzee segments, reflecting the inherent similarity of their genomic signature (see Supplementary Material Section A.5 and the confusion matrices in Supplementary Fig. S5). To address this, we repeat the experiment excluding chimpanzees from the dataset and achieve an accuracy of 91.7% using RepSeg and 91.4% using aRepSeg. In comparison, the average accuracy using random segments as representative segments across 50 runs is 84.36%. While the accuracy improves in all scenarios, the difference between the accuracy using the pipeline-selected representative and the accuracy using a random representative remains the same.

Overall, this experiment demonstrates the effectiveness of representative segment selection when choosing a portion of the genome as genome proxy for downstream analysis. In particular, taxonomic classification and clustering studies such as Arias et al.^15^ and Lichtblau et al.^30^, which rely on a random genomic segment as genome representative, or studies such as Alipour et al.^74^, which use the entire genome as representative, could benefit from our method as a preprocessing step to reliably identify a genomic segment that captures the characteristics of the whole genome. Therefore, our representative selection could improve the stability and classification/clustering accuracy of existing alignment-free taxonomic classification methods and pipelines.

## Discussion and conclusions

This study investigates the intragenomic variation of genomic signatures through the analysis of $k$-mer distributions in FCGR images and proposes effective methods to select a representative segment to serve as a proxy for the whole genome for taxonomic classification or other applications.

Overall, our findings indicate that the $k$-mer distribution reflected in FCGR images is preserved throughout the genome of the studied species, with some exceptions in repetitive regions. This counterintuitive pervasiveness of the genomic signature suggests that $k$-mer compositions within DNA sequences are shaped by fundamental patterns that tend to persist over long periods of time^12^. This being said, our analysis revealed some notable instances of FCGR patterns that deviate from the dominant genomic signature, suggesting that the pervasiveness of the genomic signature has exceptions. These exceptions, though limited in scope, may signal important biological events, and examining how and why genomic signature patterns vary in certain regions could provide valuable insights into evolutionary processes, adaptations, the detection of genomic islands, horizontal gene transfer events, and structural annotation. For instance, we hypothesize that genomic islands, which have acquired genes from other organisms, could exhibit abnormal genomic signatures compared to the host genome that might be identified through the intragenomic FCGR analysis suggested by this study. Another significant observation of this study, based on deviations in the genomic signature observed in the genomes of eukaryotic species such as human and maize, is that not all randomly selected genomic segments accurately reflect the species-specific genomic signature. This observation is especially important for downstream applications such as phylogenetic inference and sequence classification, and it highlights the significance of the proposed computational pipelines for the selection of a DNA representative genomic segment that can serve as reliable genome proxy.

This study opens several avenues for future research. Since FCGRs are $k$-mer-based representations of DNA sequences, their effectiveness can vary depending on both sequence length and the selection of parameter $k$. Given that the optimal choice of is highly dataset-dependent, with no single value consistently outperforming others across all datasets^75^, exploring adaptive or data-driven strategies for parameter selection could further improve consistency of the FCGR-based analysis^75^. Moreover, while FCGR-based methods offer a computationally efficient and alignment-free alternative to traditional approaches like BLAST^76^, they produce numerical dissimilarity scores without direct biological interpretation^36^. Future efforts could aim to correlate these quantitative measures with interpretable inferences^36^. Additionally, the representative selection pipelines and intragenomic variation experiments presented in this study have been evaluated on four complete genomes but could be extended to the study of species with potentially different characteristics, including those with bloated genomes where repetitive sequences dominate (e.g., South American lungfish, *Lepidosiren paradoxa*^77^) or those with whole-genome duplications, such as many fish lineages, where duplicated content may strongly influence FCGR-based analyses. Future research could examine the influence of specific sequence features, such as repetitive elements and structural composition, on genomic signature variation. Our preliminary masking experiments suggest that common repeats (SINEs, LINEs, LTRs, and Satellites) have only minor localized effects, but extending such analyses across species could reveal how compositional biases shape intragenomic variation. Moreover, while this study focuses on nuclear DNA, subsequent investigations may explore mitochondrial DNA (mtDNA) to assess the convergence or divergence between nuclear and mitochondrial genomic signatures. We note that extending our intragenomic variation framework to mtDNA is feasible but constrained, as it requires species with sufficiently long mtDNA sequences (such as *Silene conica* with 11 Mbp mtDNA^78^). Finally, while our experiments suggest that DSSIM is the most effective distance measure for comparing FCGRs, one could leverage machine learning to develop new distance measures that could potentially align more closely with biological factors.

Taken together, our results provide the first genome-wide, cross-kingdom assessment of how FCGR-based genomic signatures persist across complete eukaryotic genomes–and when they do not. We introduce computational and visual frameworks for intragenomic signature analysis, together with a systematic pipeline that selects a short representative segment capturing the compositional features of the whole genome. These tools could enable sensitive, spatially resolved detection of departures from the prevailing signature–such as genomic islands^79^ and horizontal gene transfers^80^–via comparisons between local segments and their genome-level representative. We further observe that the representative segment often serves as a more faithful proxy for downstream tasks than randomly chosen windows, and could improve alignment-free taxonomic classification. Overall, the work aims to move genomic signatures from a descriptive notion to an actionable object and to replace ad-hoc random sampling with representative segments–changes that could potentially yield accuracy gains and support scalable, interpretable analyses of genome-wide variation.

## Supplementary Information

Supplementary Information.

## Data Availability

The datasets generated and/or analysed during the current study are available in the https://doi.org/10.5281/zenodo.15700257 repository. Direct download links for individual datasets from NCBI are provided in the Supplementary Material.

## Code availability

All analysis code is freely available at https://github.com/Niousha12/Intragenomic_analysis.
