# Supplementary material for: Genome-wide pervasiveness and localized variation of [image]-mer-based genomic signatures in eukaryotes
Source: Sci Rep. 2026 Jun 12;16:19568. doi: 10.1038/s41598-026-40591-7 (PMC13294376; doi:10.1038/s41598-026-40591-7)
Supplement: Supplementary file 1 — Supplementary Information. [file 41598_2026_40591_MOESM1_ESM.pdf]

Supplementary Information for

Genome-wide Pervasiveness and Localized Variation of k-mer-based Genomic Signatures in Eukaryotes

Niousha Sadjadi, Camila P. E. de Souza, Gurjit Randhawa,

Kathleen A. Hill, and Lila Kari

Overview

This document provides supplementary information for the main report titled "Genome-wide Pervasiveness and Localized Variation of k-mer-based Genomic Signatures in Eukaryotes." It includes additional figures, tables, and technical details.

A Detailed Methods

A.1 Distance Measures Analyzed

In the calculation of vector-based distances, x_i represents the i-th position in the FCGR vector X, y_i represents the i-th position in the FCGR vector Y, and L denotes the length of the vectors, which is equal to the total number of pixels in an FCGR image.

Normalized Euclidean distance is a vector-based distance and is the normalized derivation of the Euclidean distance that is more suitable for comparison across different scales. In our study, to compute the Normalized Euclidean distance between two FCGR vectors, we use the following equation:

$$d_{\text{Normalized Euclidean}}(X,Y) = \frac{\sqrt{\frac{1}{L}\sum_{i=1}^{L}(x_i - y_i)^2}}{\sqrt{\frac{1}{L}\sum_{i=1}^{L}(x_i)^2}}. \quad (1)$$

In this formulation, one of the FCGR images is used as the reference for normalization. A disadvantage of this distance when comparing two FCGRs is its lack of symmetry; specifically, $d_{\text{Normalized Euclidean}}(X,Y)$ is not necessarily equal to $d_{\text{Normalized Euclidean}}(Y,X)$. To address this issue, we compute the Euclidean norm (i.e., the denominator of Eq. 1) for both FCGRs and use the image with the larger value as the reference. A value of 0 in Normalized Euclidean distance indicates that the two images are identical, while a value in the range $(0,1]$ indicates that the two images are somewhat different, with the error being within the

range of the reference image's magnitude. However, a value greater than 1 indicates that the two images are significantly different, with the error exceeding the range of the reference image's magnitude.

Cosine distance, which is another vector-based distance, is calculated by subtracting the cosine similarity of two FCGR vectors from 1. Considering that cosine similarity takes a value between $[-1, 1]$, the cosine distance theoretically varies between $[0, 2]$. In order to calculate the cosine similarity and then cosine distance we can use the following equations:

$$\begin{aligned} \text{Cosine\_Similarity}(X, Y) &= \frac{\sum_{i=1}^{L}(x_i \cdot y_i)}{\sqrt{\sum_{i=1}^{L}(x_i)^2} \cdot \sqrt{\sum_{i=1}^{L}(y_i)^2}}, \\ d_{\text{Cosine}}(X, Y) &= 1 - \text{Cosine\_Similarity}(X, Y). \end{aligned} \tag{2}$$

Manhattan distance is another vector-based distance measure. It is computed as the sum of the absolute differences between corresponding elements of two FCGR vectors, and it can theoretically range from 0 to ∞. However, in our case, the upper bound is limited to $2^k \times 2^k$ due to the resolution of the FCGRs, where k represents the value used in the k-mer. The Manhattan distance between two FCGR vectors is given by:

$$\text{Manhattan}(X, Y) = \sum_{i=1}^{L} |x_i - y_i|. \tag{3}$$

Structural Dissimilarity Index (DSSIM), is calculated by subtracting Structural Similarity Index (SSIM) from 1. SSIM is a measure used to quantify the similarity between two images in three main aspects: luminance, contrast, and structure [23]. Luminance comparison measures the difference in the mean intensity or brightness of the images, contrast comparison measures the differences in the standard deviation or contrast of the images, and structure comparison measures the correlation between the local structures and patterns of the images. The SSIM between two images X and Y combines these three comparisons into a single score:

$$\text{SSIM}(X, Y) = [l(X, Y)]^{\alpha} \cdot [c(X, Y)]^{\beta} \cdot [s(X, Y)]^{\gamma}, \tag{4}$$

where $l(X, Y)$ is the luminance comparison, $c(X, Y)$ is the contrast comparison, and $s(X, Y)$ is the structure comparison function between the two images. The parameters α, β, and γ are used to adjust the importance of each term.

Luminance measures the brightness level of an image. The luminance comparison function evaluates how the mean intensity (brightness) values of the two images differ. Given two images X and Y, the luminance comparison function is defined as:

$$l(X, Y) = \frac{2\mu_X \mu_Y + C_1}{\mu_X^2 + \mu_Y^2 + C_1}, \tag{5}$$

where μ_X is the local mean intensity matrix of image X, μ_Y is the local mean intensity matrix of image Y, and C_1 is a small constant to avoid division by zero. Typically, $C_1 = (K_1 L)^2$, where L is the dynamic range of the pixel values and K_1 is a small constant, e.g., 0.01. The local mean of image X is computed as:

$$\mu_X = G \otimes X, \tag{6}$$

where X is the input image and G is a two-dimensional Gaussian kernel with a variance of 1.5 that is convolved with X. Thus, μ_X is a matrix of the same size as the input image, containing a blurred version of X.

The function $l(X, Y)$ is designed to be 1 when the luminance of the two images is the same and decreases as the luminance difference increases.

Contrast measures the difference in the intensity values within an image, reflecting how varied the pixel values are from the mean. The contrast comparison function between two images X and Y is defined as:

$$c(X, Y) = \frac{2\sigma_X \sigma_Y + C_2}{\sigma_X^2 + \sigma_Y^2 + C_2}, \tag{7}$$

where σ_X and σ_Y are the local standard deviation of the corresponding images X and Y, which represent the image contrast. Additionally, C_2 is another small constant to avoid division by zero. Typically, $C_2 = (K_2 L)^2$, where K_2 is another small constant, e.g., 0.03, and L is the dynamic range of the pixel values.

To compute the local standard deviation of an image, we can convolve a Gaussian kernel filter using the following formulation:

$$\sigma_X^2 = G \otimes (X - \mu_X)^2, \tag{8}$$

where G represents the Gaussian kernel filter and $\otimes$ denotes convolution. This function is designed to be 1 when the contrast of the two images is the same and decreases as the contrast difference increases.

Structure measures the correlation between the patterns of pixel intensities in the images, reflecting how similar the local structures are. The structure comparison function between two images X and Y is defined as:

$$s(X, Y) = \frac{\sigma_{XY} + C_3}{\sigma_X \sigma_Y + C_3}, \tag{9}$$

where σ_{XY} is the local covariance of images X and Y, representing how well the pixel intensities in one image can be predicted by the pixel intensities in the other. C_3 is a small constant, typically $C_3 = C_2/2$.

The local covariance can be computed by convolving the product of the deviations from the mean of the two images with a Gaussian kernel filter:

$$\sigma_{XY} = G \otimes ((X - \mu_X)(Y - \mu_Y)), \tag{10}$$

where G represents the Gaussian kernel filter and $\otimes$ denotes the convolution operation. This function is designed to be 1 when the structural patterns of the two images are perfectly correlated and decreases as the correlation weakens.

The overall SSIM is computed by combining these three components as mentioned in Equation 4. To obtain the structural dissimilarity index (DSSIM), we subtract the SSIM from 1. SSIM values range from -1 to 1, where 1 indicates perfect similarity between the images, 0 indicates no similarity, and values less than 0 indicate structural dissimilarity. Consequently, the SSIM distance (d_{SSIM}), defined as $1 - \text{SSIM}$, ranges from 0 (for identical images) to 2 (for images that are completely dissimilar or negative of each other).

Descriptor distance between two images refers to a measure of similarity or dissimilarity based on specific features extracted from those images. After the features are extracted, they are typically converted into a numerical vector known as descriptors. The distance is then computed as the Normalized Euclidean distance between the corresponding descriptor vectors of the two images. In the case of FCGR comparison, to obtain the descriptor vector, we follow hierarchical image descriptors suggested by [8, 9].

For two FCGR images X and Y, where $X, Y \in \mathbb{N}^{2^k \times 2^k}$, we calculate the distance as follows: first, we split each of the two images into non-overlapping sub-images of size $2^m \times 2^m$, where m ($m < k$) is a hyperparameter of the distance measure [8]. After this division, we have $2^{2(k-m)}$ submatrices X_{ij} and Y_{ij} with $i, j = \{1, \ldots, 2^{k-m}\}$ [8]. To create the descriptor vector for a sub-image X_{ij}, we first divide the range of possible pixel values in an FCGR image into r bins. These bins are defined as $[0, k_1), [k_1, k_2), \ldots, [k_{r-1}, \infty)$. Next, we construct a vector of length r, $(b_1, b_2, b_3, \ldots, b_r)$, where each component b_z represents the total number of pixels within the sub-image X_{ij} whose values fall into the corresponding bin $[k_{z-1}, k_z)$. Therefore, the extracted features from the images are local histograms with predefined bins. The final descriptor vector (DV) for the whole FCGR image is obtained by concatenating the descriptor vectors of all sub-images [8]. Finally, we use the following formulation for calculating the descriptor distance in our study:

$$d_{\text{Descriptor}}(X, Y) = \text{Normalized Euclidean}(\text{DV}(X), \text{DV}(Y)). \tag{11}$$

Similar to Normalized Euclidean, a descriptor distance of 0 means that the descriptor vectors of two images are completely identical, while values greater than 1 indicate a significant distance between the descriptor vectors.

Learned Perceptual Image Patch Similarity (LPIPS) is a deep learning-based method for measuring the similarity and distance between two images [26]. This method utilizes the intermediate feature space of a convolutional neural network to produce a distance measure that considers both structural and perceptual similarities of two images.

The input image passes through all these layers sequentially, with each layer extracting specific features. These layers are trained to recognize high-level features and textures in images. The early layers focus on structural features, while the later layers capture more perceptual aspects. To calculate the distance, LPIPS first computes the Euclidean distance between the feature maps of the two input images at corresponding layers. These layer-wise distances are then weighted and summed to produce the final distance score.

To compute the LPIPS distance for two FCGR images, the same process is applied. Equation 12 shows the formulation for the LPIPS distance. In this equation, X and Y represent the two FCGR images, $\hat{\phi}^l$ denotes the normalized feature map at layer l extracted using the pre-trained network, and w^l represents the learned weights used to combine the distances from M different layers.

$$d_{\text{LPIPS}}(X, Y) = \sum_{l=1}^{M} w^l \|\hat{\phi}^l(X) - \hat{\phi}^l(Y)\|_2. \tag{12}$$

Kolmogorov-Smirnov (K-S) distance is based on the non-parametric test statistic that compares the distributions of two univariate datasets to determine if they differ significantly. As discussed earlier, one way to conceptualize FCGR representations is to view

them as distributions of different k-mers. We can treat FCGRs as probability distributions of k-mers, allowing us to apply statistical methods to compare two FCGRs. This approach leverages the principles of probability and statistics to analyze and measure the similarities and differences between the FCGR representations. Therefore, we consider the K-S test statistic as a measure of distance between two FCGR images.

For two FCGR images $X, Y \in \mathbb{N}^{2^k \times 2^k}$, we initially convert each of them to one-dimensional vectors. Each vector is then converted to probability values by dividing each position value by the sum of all values. Next, we calculate the cumulative distribution function (CDF) of the probabilistic FCGRs. Finally, we return the maximum value over all possible values in the absolute subtraction of the CDF of the two images. The equation is:

$$d_{\text{K-S}}(X, Y) = max_z |F_X(z) - F_Y(z)|. \tag{13}$$

In this formulation, $F_X(z)$ is the cumulative distribution function (CDF) of the probabilistic FCGRs at position z. The range of the K-S distance is $[0, 1]$. A value of 0 indicates that the empirical distribution functions of the two samples are identical, while a value of 1 indicates the maximum possible difference between the empirical distribution functions.

Wasserstein distance is a non-parametric method for comparing the distributions of two datasets to determine their similarity, analogous to the K-S test. More specifically, the Wasserstein distance measures the minimum amount of “work” required to transform one distribution into another. In this context, “work” is defined as the amount of distribution mass that needs to be moved multiplied by the distance it needs to be moved.

To apply the Wasserstein distance between two FCGR images X and Y, similar to the K-S test, we first flatten each FCGR image into a vector, then calculate the probability values, and subsequently utilize the cumulative distribution function (CDF). Finally, we use the following equation to compute the distance:

$$d_{\text{Wasserstein}}(X, Y) = \sum_z |F_X(z) - F_Y(z)| \, dz, \qquad \text{where } dz = \frac{1}{L}. \tag{14}$$

In this formulation, $F_X(z)$ represents the cumulative distribution function (CDF) of the probabilistic FCGR X at position z, and L is the total number of positions or pixels.

The Wasserstein distance ranges from 0 to 1. A distance of 0 indicates that the two distributions are identical, meaning no “work” is needed to transform one distribution into the other. Conversely, a distance of 1 indicates that the two distributions are completely different, meaning they are as far apart as possible at every point z.

Before calculating the values of different distance measures on the FCGR images, we apply a preprocessing step to each of our distance groups. For image-based and vector-based distance measures, we apply the min-max normalization to rescale the FCGR values to the $[0, 1]$ range, thereby enhancing the comparability of two FCGR images. The effectiveness of this normalization is well-documented in the literature, as it manages the large variance in FCGR images [12] and ensures that all elements contribute equally to the distance calculations [4]. A key advantage of this normalization in FCGR comparison is that it enables the comparison of FCGRs derived from sequences of different lengths. The normalization process for these two distance groups is applied as follows:

$$\text{NormalizeFCGR}(X) = \left\{ \frac{x_i - \min(X)}{\max(X) - \min(X)} \mid x_i \in X \right\}. \tag{15}$$

For probability-based distances, such as the K-S test and Wasserstein distances, which use statistical methods operating on probability distributions, we apply probability normalization to the FCGRs. This ensures that the sum of all values equals one before performing these tests. The normalization process is applied as follows:

$$\text{NormalizeFCGR}(X) = \left\{ \frac{x_i}{\sum_{j=1}^{n} x_j} \mid x_i \in X \right\}. \tag{16}$$

A.2 Experiments for Human Intragenomic Distance Analysis (Exp 2.1)

The detailed description of the experiments conducted for the *Human Intragenomic Distance Analysis* are as follows:

- *Telomere vs. Telomere*: This experiment compares the distance between telomeric regions of different chromosomes. Telomeres are identical short sequence tandem repeats located at the ends of the p-arms and q-arms of all of the chromosomes [19]. The composition and structure of telomeres are known to be similar across different human chromosomes, and these similarities are attributed to the conserved nature of telomeric DNA sequences and the associated proteins that form these protective structures [11]. Human telomeres typically consist of thousands of short 'TTAGGG' tandem repeats, which vary in length with age, leading to telomere lengths that typically range from 5 to 15 Kbp [25]. These repeats are crucial for maintaining chromosome stability and integrity [11]. In this experiment, we calculate the average distance between the p-arm telomere of the first human chromosome and the p-arm telomeres of the other chromosomes. This approach can be applied to both p-arm and q-arm telomeres; however, for consistency, we choose to focus on the p-arm telomere. Similarly, this experiment could be extended to centromeres, as they also contain highly repetitive sequences of identical repeats [6], known as alpha-satellite DNA [21]. These alpha-satellite monomers are approximately 171 bp in length [21]. However, given the expected similarities in centromere comparisons, we focus exclusively on telomeres.

- *Heterochromatin vs. Heterochromatin*: Heterochromatin in all chromosomes consists of highly condensed, repetitive, and transcriptionally inactive regions that show similarities across different chromosomes [22, 16]. These regions are represented by black and three shades of gray in the NCBI Data Viewer, where darker shades correspond to more condensed chromatin, reflecting higher levels of chromatin compaction and staining intensity [5]. In this experiment, we initially extract the most condensed regions of different chromosomes, which are colored in black. For chromosomes 16 and 21, where only one black region is present, we also include the dark gray region. For chromosomes 15, 17, 19, 20, 22, and Y, where there are no black regions, we select the next most condensed region, represented by other shades of gray. For each chromosome, we calculate the average distance between the most condensed heterochromatic region distal to the centromere on the p-arm (or, if none exists, proximal to the centromere on the q-arm) and other heterochromatic regions within the same chromosome. We then report the average distance across all chromosomes and evaluate the performance of different distance measures.

- *Heterochromatin vs. Euchromatin*: Euchromatin describes regions of DNA that are less condensed and contain genes that are actively transcribed into RNA [1]. Euchromatin and

heterochromatin regions differ significantly in their overall structure and function [1]. Euchromatin is associated with gene-rich regions and active transcription, while heterochromatin is linked to gene-poor regions and gene silencing [1]. Euchromatin has higher C+G content and CpG density, associated with active gene transcription, while heterochromatin has lower C+G content and fewer, often methylated, CpG sites, leading to gene silencing [14]. In this experiment, for each chromosome, we first measure the distance between the most condensed heterochromatic region distal to the centromere on the p-arm (or, if none exists, proximal to the centromere on the q-arm) and each of four randomly selected euchromatic segments. We then report the average distances across all chromosomes for comparison between the distance measures.

- *p-arm vs. q-arm (for acrocentric chromosomes)*: Acrocentric chromosomes are distinguished by their centromere being positioned very close to one end, resulting in a short p-arm and a long q-arm [20]. The short arm of these chromosomes is a stretch of DNA sequence that contains tandem repeat sequences, while their long arm contains less of these tandem repeat sequence arrays [13]. Large tandem repeats in chromosomes are DNA sequences composed of multiple copies of a particular sequence arranged in a head-to-tail (tandem) manner [3]. Inspired by the structural differences between the p-arm and q-arm of acrocentric chromosomes, we design this experiment using the acrocentric chromosomes in humans, which are the five autosomal chromosomes 13, 14, 15, 21, and 22 [13], as well as chromosome Y [2]. The repetitive region on the Y chromosome is located on the long arm (q-arm) and is fundamentally different in overall length and the length and nature of the tandem repeat arrays [2]. In our experiment, we examine each of these chromosomes individually. For each chromosome, we randomly select a 500 Kbp segment from the p-arm and another 500 Kbp segment from the q-arm, then calculate the distance between them. This process is repeated 100 times to ensure variability and randomness in segment selection, and the results are then averaged.

- *Large Tandem Repeat Arrays*: Large tandem repeats, classified as moderately repetitive structures [10], vary significantly in size and the number of repeat units and are categorized based on their length and organization [24]. These repeats can be found in the p-arm of acrocentric chromosomes and the q-arm of chromosome Y. However, the large tandem repeat arrays in the q-arm of chromosome Y differ in both length and structural composition from those in acrocentric chromosomes [18]. In this intragenomic experiment, we compare the q-arm of the Y chromosome to the large tandem repeat arrays of the acrocentric chromosomes by calculating the distance between the cytoband q12 of chromosome Y and each of the cytobands containing large tandem repeat arrays on chromosomes 13, 14, 15, 21, and 22. The average of these distances is then reported. The q12 cytoband on the Y chromosome is approximately 35 Mbp in length, which is part of the total 62 Mbp of the Y chromosome. The approximate lengths of the large tandem repeat arrays are as follows: for chromosome 13, 16 Mbp out of 114 Mbp; for chromosome 14, 10 Mbp out of 101 Mbp; for chromosome 15, 17 Mbp out of 100 Mbp; for chromosome 21, 11 Mbp out of 45 Mbp; and for chromosome 22, 13 Mbp out of 51 Mbp. Compared to the *p-arm vs. q-arm* experiment, which contrasts tandem repeats with non-repeats, the *Large Tandem Repeat Arrays* experiment focuses on comparing different types of tandem repeat regions.

- *Arbitrary Sequences*: In the final experiment, we aim to determine an intermediate intragenomic distance between FCGRs. To do so, we randomly select two non-overlapping 500 Kbp sequences from a randomly chosen chromosome and compute the distance between their FCGRs. This process is repeated 100 times, and the average distance is used for comparison. Given the 100 repetitions of this random selection process, we expect it to sample diverse combinations of genomic sequences, including tandem repeat arrays and non-repetitive regions.

A.3 Effect of Sequence Length and k-mer Size on Classification Performance

A classification experiment is conducted similar to Exp 4 in the manuscript, except that both the sequence length and the value of k systematically varied to evaluate their effects on classification performance (for details of the classification test, see *Experimental Design – Exp 4* in the manuscript). The values of k ranged from 3 to 8, and the sequence lengths were set to 100 Kbp, 200 Kbp, and 500 Kbp. Due to computational constraints of FCGR generation, additional values could not be included. The results of this experiment are presented in Table S1.

As shown in Table S1, k-mer sizes of 4, 5, and 6 yield better classification performance compared to other values. However, smaller k values such as 4 and 5 produce FCGR images with limited visual resolution. Therefore, $k = 6$ represents an optimal balance between classification performance and visual resolution. This choice is also biologically meaningful, as 6 is a multiple of codon length.

Regarding sequence length, both 200 Kbp and 500 Kbp segments achieve comparable performance. These lengths are used interchangeably in different experiments depending on experimental constraints.

A.4 Effect of Repetitive Elements on Intragenomic Variation

To examine how repetitive elements such as SINEs, LINEs, LTRs, and Satellites contribute to intragenomic variation, an analysis is performed on human chromosomes 1, 9, 15, 16, and Y, in which specific repeat classes are selectively masked. First, following the same configuration as Exp 3.1, each chromosome is divided into consecutive non-overlapping segments of 500 Kbp, the representative segment is identified using the RepSeg method, and DSSIM distance is calculated between the FCGR of each segment and that of the representative segment ($k = 6$ is used throughout the analysis). Then, the same analysis is repeated after independently masking each repeat class by replacing the annotated positions of SINEs, LINEs, LTRs, and Satellites with 'N' while preserving sequence length (annotations for these elements are obtained from the RepeatMasker [17]). For each masked version, new FCGRs are generated for all segments, and DSSIM distances to the masked representative segment are recomputed. The original representative segment, identified from the unmasked genome using RepSeg, is kept fixed across all masking experiments and is not reselected after masking. However, any repeat regions within it are replaced with 'N' to maintain consistency with the masked genome. Fig. S7 shows the intragenomic variation profiles for the unmasked sequence (red), masked sequence (green), and their difference (blue) for each type of repeat.

To quantify the changes introduced by repeat masking, Table S6 reports the number of segments with a DSSIM distance below 0.24 from the representative segment and the mean DSSIM distance (from the intragenomic variation plot), as well as the mean squared error (MSE) between the unmasked and masked intragenomic variation plots for each repeat class.

Comparing the unmasked and masked intragenomic variation allows us to evaluate how much each repeat class influences the intragenomic variation of the genomic signature along the chromosome. The results in Fig. S7 and Table S6 indicate that masking SINEs, LINEs, and LTRs has variable local effects on intragenomic variation, reducing the distance from the representative segment in some regions while increasing it in others. However, in both chromosome 1 (the largest human chromosome) and chromosomes 9, 15, and 16 (those with the highest variability), masking these interspersed repeats has only a negligible influence ($<\pm 0.05$) on the average DSSIM distance. Satellites are mostly concentrated in centromeric regions or the acrocentric p-arms, so their masking primarily affects these localized areas and has minimal impact on the remainder of the chromosome. In some chromosomes, portions of the centromeric regions consist almost entirely of Satellite sequences, so once masked and replaced with stretches of 'N's, these regions cause discontinuities in the intragenomic variation plots shown in Fig. S7. Despite these localized effects, masking Satellites—similar to masking SINEs, LINEs, and LTRs—does not substantially alter the mean DSSIM distance. Chromosome Y is an outlier among the human chromosomes, in that it has a highly repeated structure (its entire q-arm), and these repeats influence the chromosome-representative segment selection. Consequently, unlike the other chromosomes, the chromosome-Y-representative segment is drawn from its repeat-rich q-arm. Therefore, masking Satellites masks approximately 35% of the representative segment itself, which is not observed in any other chromosome, while masking SINEs, LINEs, and LTRs has a negligible impact on the segments within the q-arm.

Overall, masking SINEs, LINEs, LTRs, and Satellites does not substantially alter the intragenomic distance variability in the analyzed chromosomes. Although minor localized effects are detectable, the underlying signature remains consistent and is not driven by these interspersed repetitive elements, indicating that they are not major contributors to either the genomic signature itself or its intragenomic variation. The substantial variation observed in the genomic signature primarily arises in the centromeric regions or the acrocentric p-arms due to their different k-mer composition, while the few localized regions of high distance are very few in number and they are observed with and without repeat masking. These regions have notable sequence features such long noncoding DNAs which have reduced G and C content and GC skew.

A.5 Effect of Repeat Masking on Human–Chimpanzee Classification

The confusion matrix in Fig. S5 shows that most classification errors in Exp 4 occur between the human and chimpanzee classes. To examine the effect of each repeat class on this challenging classification, an experiment similar to Exp 4 was performed using only the first chromosomes of the human and chimpanzee genomes (which are homologous). The experiment is first conducted on the unmasked sequences and then reapplied after independently

masking each repeat class by replacing the annotated positions of SINEs, LINEs, LTRs, and Satellites with 'N' while preserving sequence length (annotations are obtained from the corresponding RepeatMasker [17] for chromosome 1 of both species). Table S7 reports the classification accuracies for each masking condition.

As indicated in Table S7, masking these repeat classes does not noticeably affect the classification accuracy. This finding aligns with the earlier observation that repetitive elements do not substantially influence the genomic signature itself. The genomic signatures of human and chimpanzee are structurally similar regardless of the presence of these repeats, and since the repeats are not major contributors to the signature, masking them does not alter the outcome of the classification experiment.

B Supplementary Figures

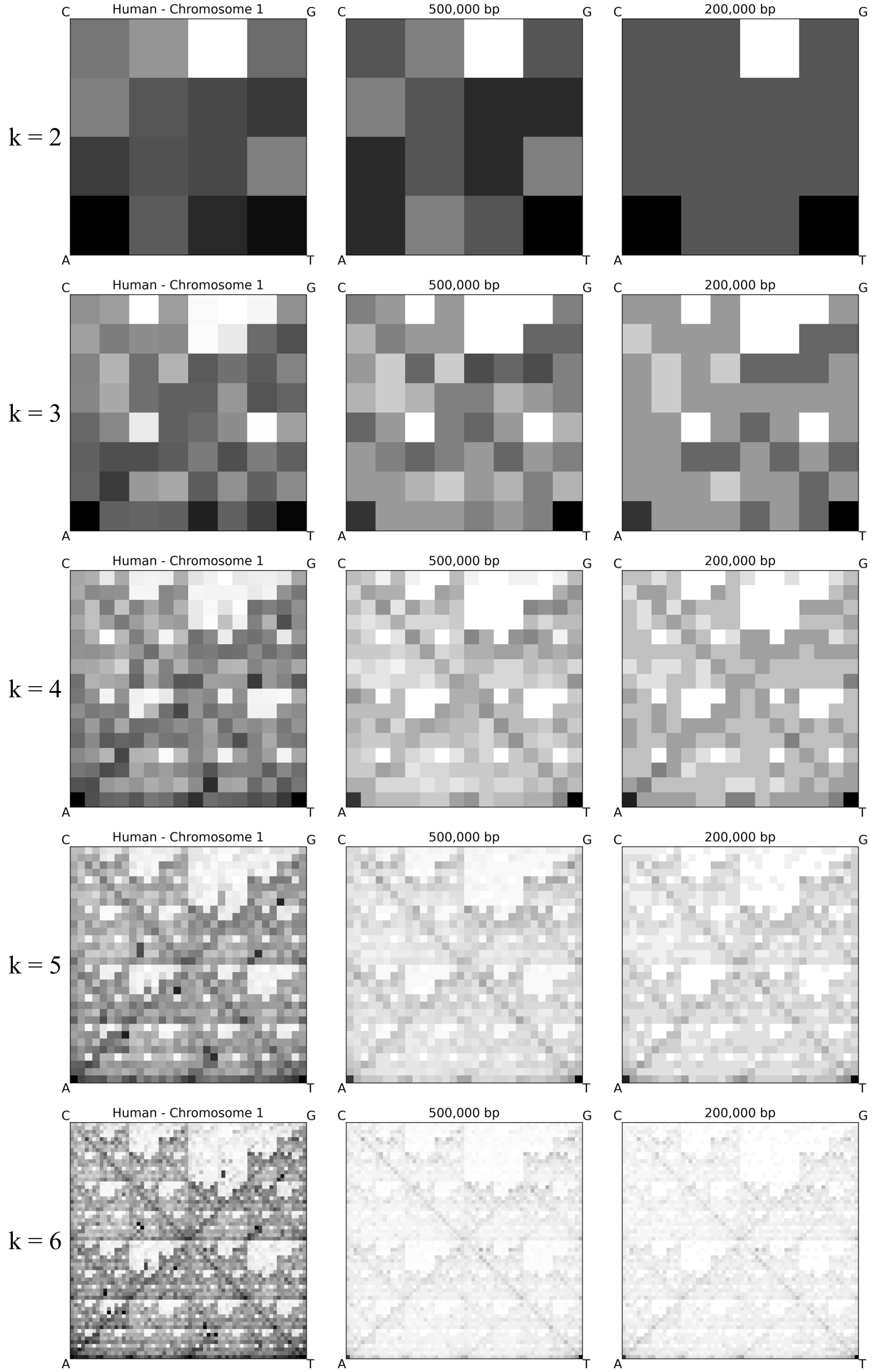

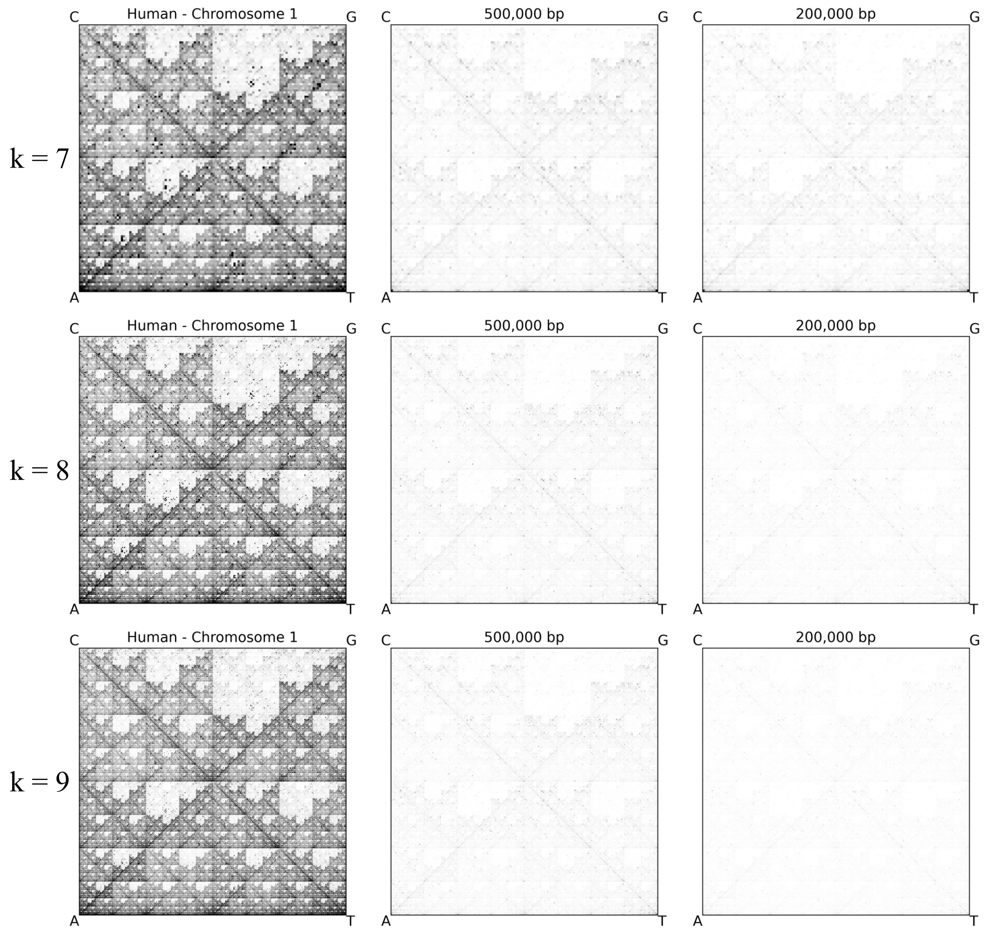

Figure S1: **Effects of k-mer size and sequence length on the resolution of FCGR images.** The first column shows the FCGR of the complete human chromosome 1 (248 Mbp). The second column corresponds to a 500 Kbp segment from the beginning of cytoband p34.1 (a euchromatic, gene-rich region), and the third column represents a 200 Kbp segment from the same region. For shorter sequences, increasing the k-mer size enhances resolution but results in fainter images. When sequence lengths are sufficiently long, higher k values yield both high resolution and visually rich patterns, effectively capturing genomic signatures. Note that the visualization scaling used in this analysis, for consistency among values of k, is slightly different from the visualization used in Fig. 5 of the manuscript, but this does not affect the quantitative analysis.

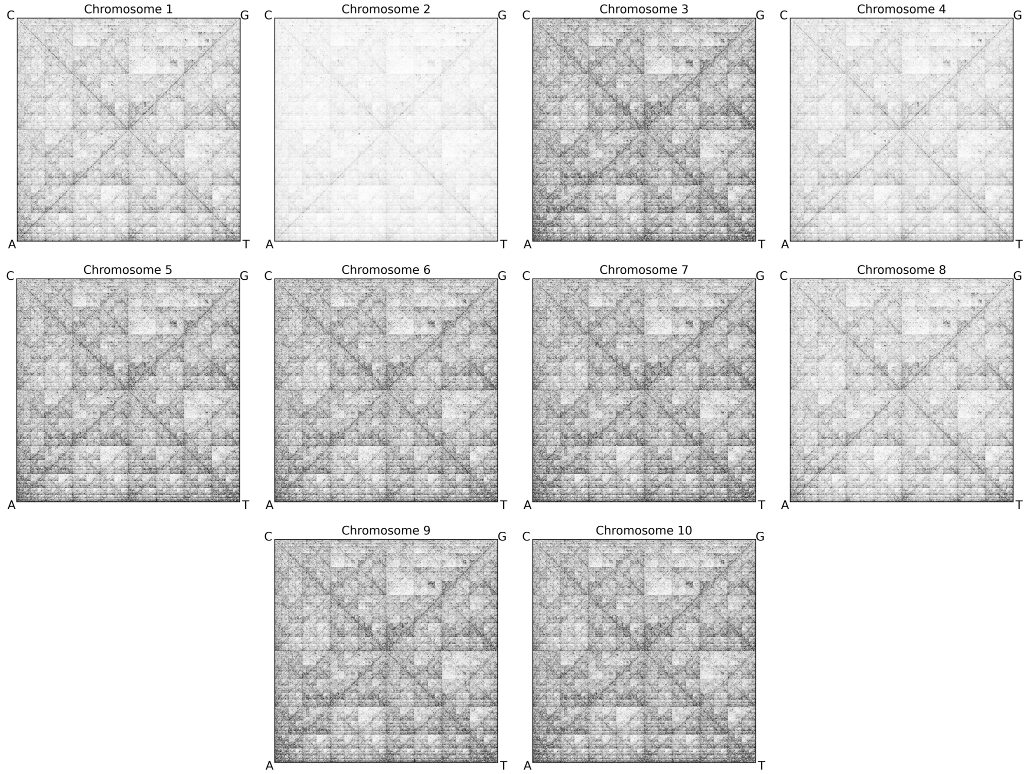

Figure S2: **Analyses of the Maize Genome Genetic Signature.** Each image displays the FCGR of a complete maize chromosome, generated using $k = 9$. The patterns reveal both conserved genomic features and variations in k-mer distribution, indicating that while the overall structure is largely consistent across chromosomes, differences in shading highlight regions with uneven k-mer composition. Notably, chromosomes 2 and 4 appear paler, suggesting the presence of highly repeated k-mers (9-mers in this case) within their sequences.

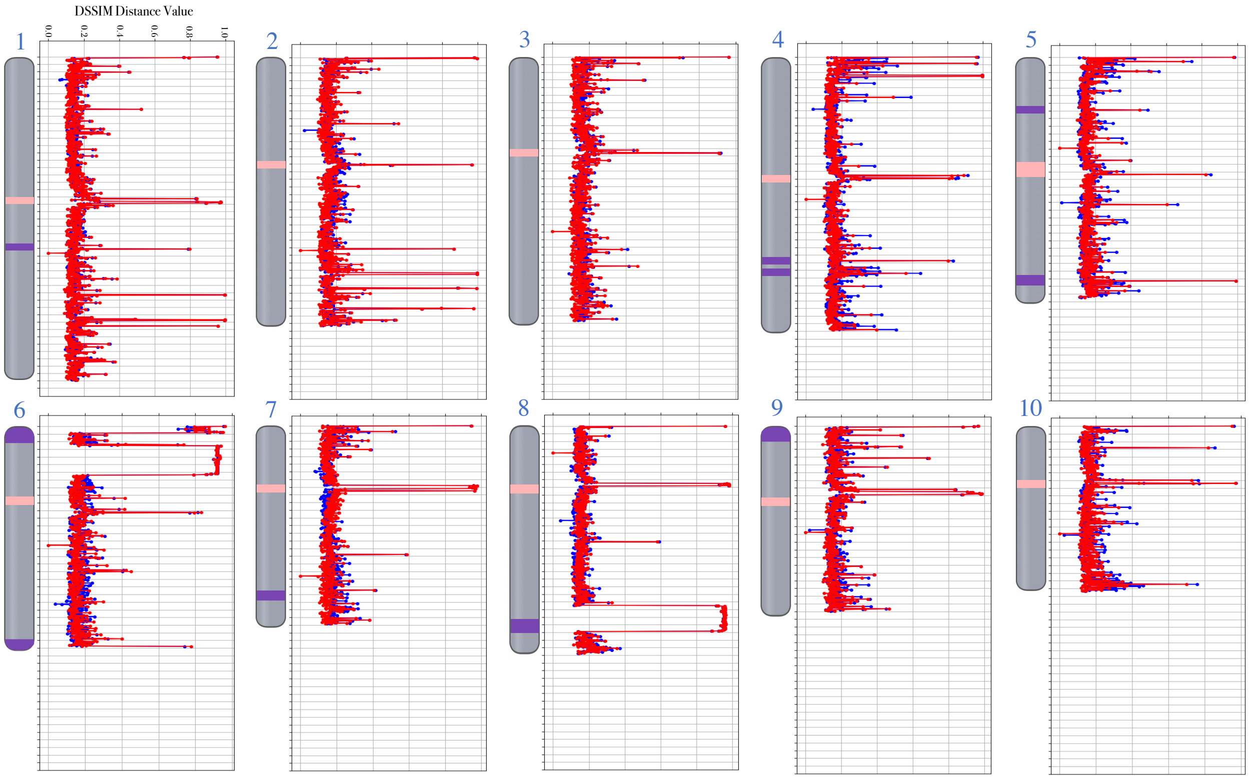

Figure S3: **DSSIM distances of consecutive segments from the representative segment across all maize chromosomes.** The red and blue line plots represent Exp 3.1 and Exp 3.2, respectively. Each point corresponds to the calculated distance between a 500 Kbp genomic segment and the representative segment selected by the proposed pipelines. The horizontal axis displays DSSIM distance values ranging from 0 to 1 in increments of 0.2, where higher values indicate greater dissimilarity. The vertical axis is segmented into intervals of 20, corresponding to sequential 500 Kbp segments along each chromosome. To the left of each plot, chromosome ideograms are generated using the NCBI Genome Data Viewer [15], and the suggested centromere and KNOB180 region annotations by Hufford et al. [7]. These ideograms provide an approximation of the positions of centromeres (pink regions) and tandem repeat arrays known as KNOB180 (purple regions).

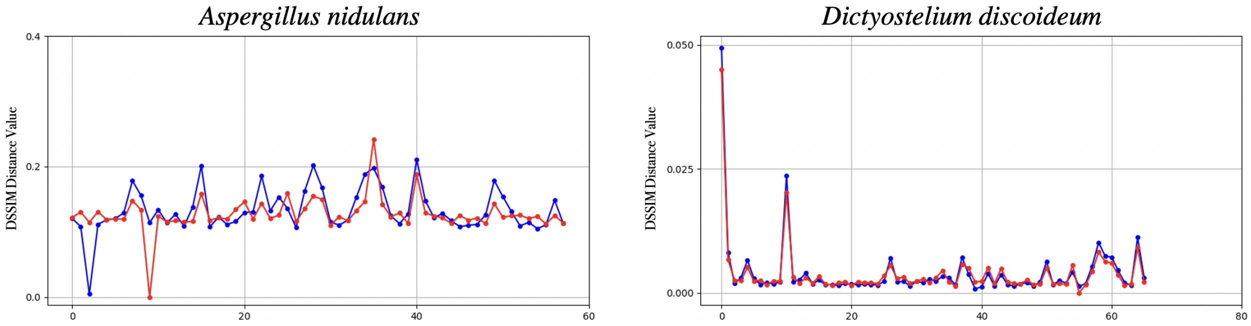

Figure S4: **DSSIM distances of consecutive segments from the representative segment for two eukaryote species (left: *Aspergillus nidulans*, right: *Dictyostelium discoideum*).** The red line plots show the DSSIM distances between consecutive 500 Kbp segments and the representative segment identified by RepSeg, while the blue line plots illustrate the DSSIM distances from the representative segment selected by aRepSeg. The horizontal axis is segmented into intervals of 20, representing sequential 500 Kbp segments along each chromosome, while the vertical axis shows the DSSIM distance values. Notably, the vertical axis range differs between the two species to accommodate their respective DSSIM distance distributions. In *Aspergillus nidulans*, DSSIM distances are higher, reaching up to 0.4, whereas in *Dictyostelium discoideum*, distances remain much lower, with a maximum of 0.05.

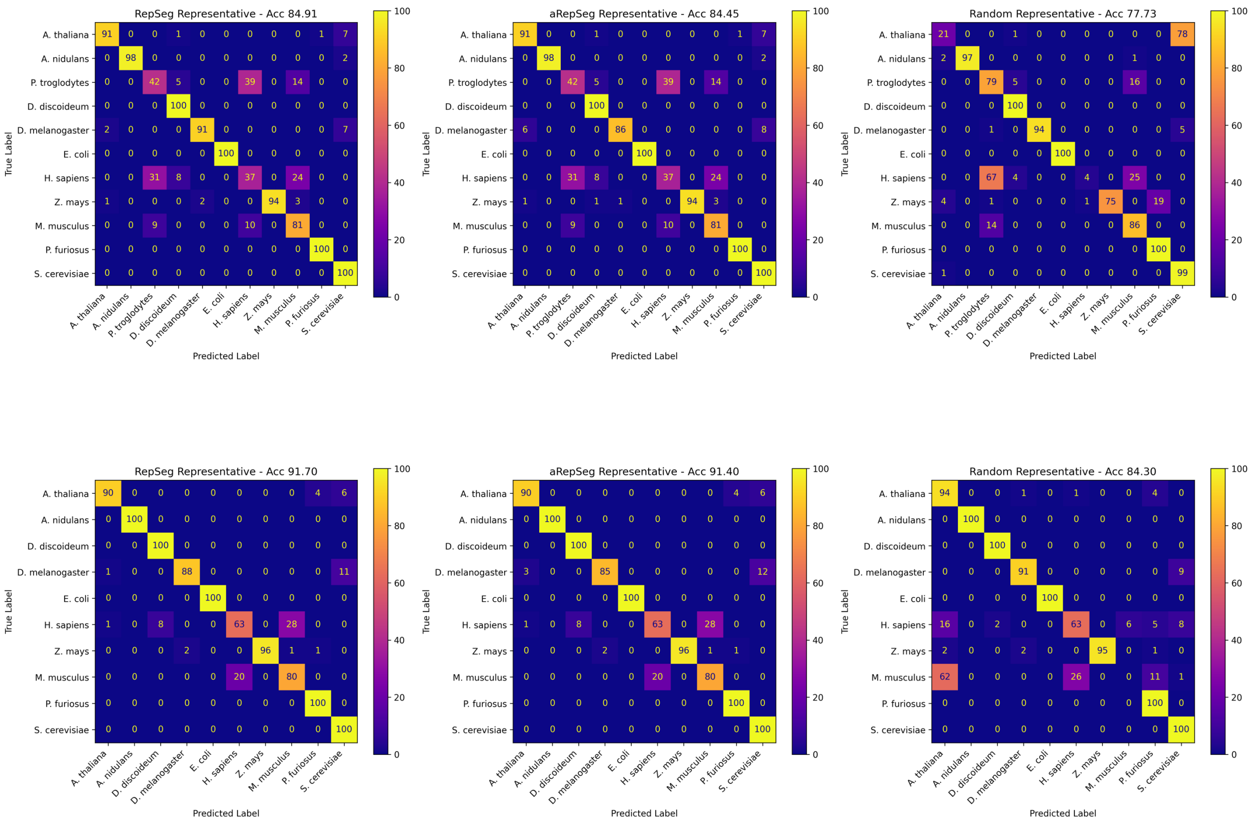

Figure S5: **Confusion matrices for the nearest neighbor classifier (Experiment 4).** The top row displays confusion matrices for the experimental setup that includes all species from the study. Due to the high genetic similarity between the human genome and chimpanzee genome, most misclassifications occur between these two species. The bottom row illustrates the results when the chimpanzee species is excluded from the test, leading to improved classification performance. Notably, while the overall accuracy increases in this scenario, the difference in accuracy between the pipeline-selected representative and the random representative remains the same. Among the approaches, the RepSeg method (left) achieves the highest accuracy, with aRepSeg (middle) performing similarly well, whereas the random representative (right) is less optimal in comparison.

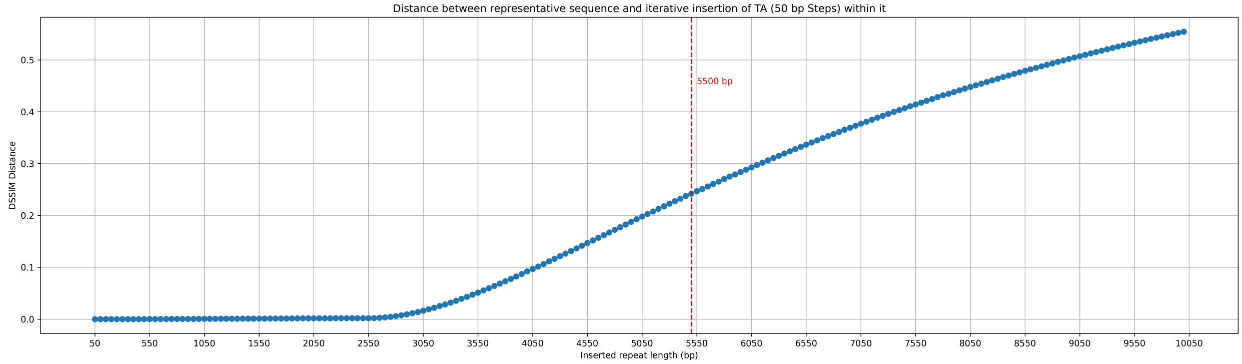

Figure S6: **Effect of iterative insertion of repetitive sequence on DSSIM distance.** The representative segment (500 Kbp) of human chromosome 1 was progressively modified by inserting the dinucleotide repeat "TA" in 50 bp increments at a fixed random start position. The plot shows the DSSIM distance between the original and modified sequences as the inserted repeat length increases. The red dashed line marks the point (5.5 Kbp) where the DSSIM distance first reaches 0.24, corresponding to only 1.1% of the total sequence being replaced. This result highlights that a relatively small localized modification is sufficient to produce a DSSIM distance of 0.24.

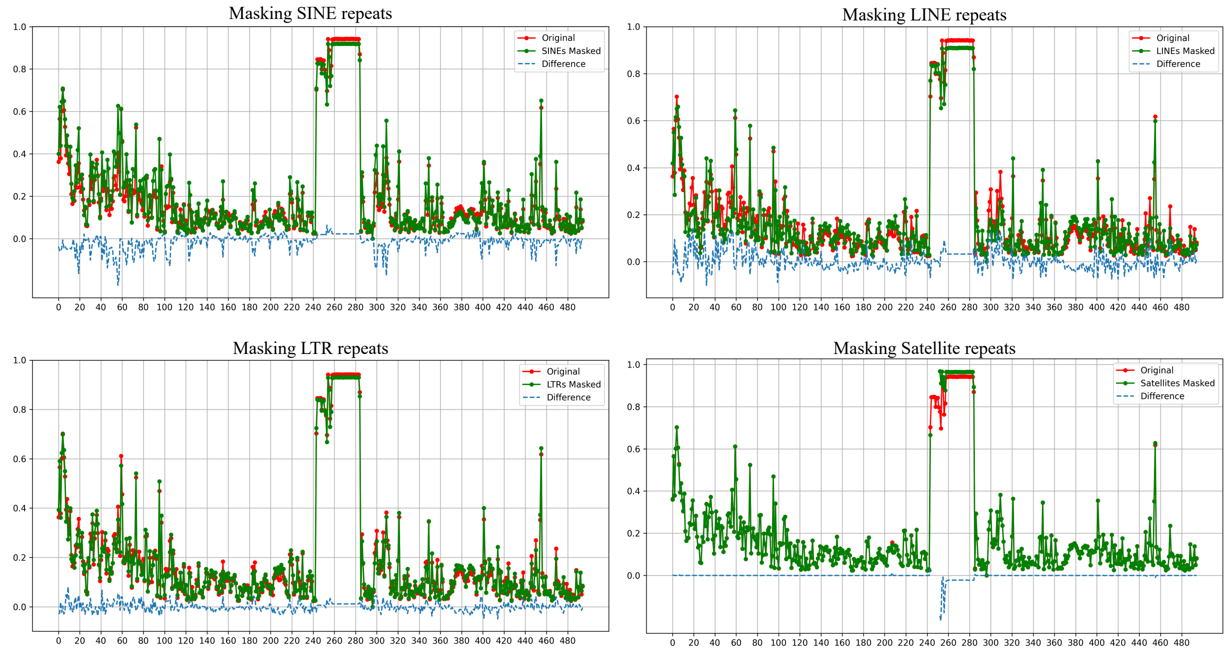

(a) Human chromosome 1.

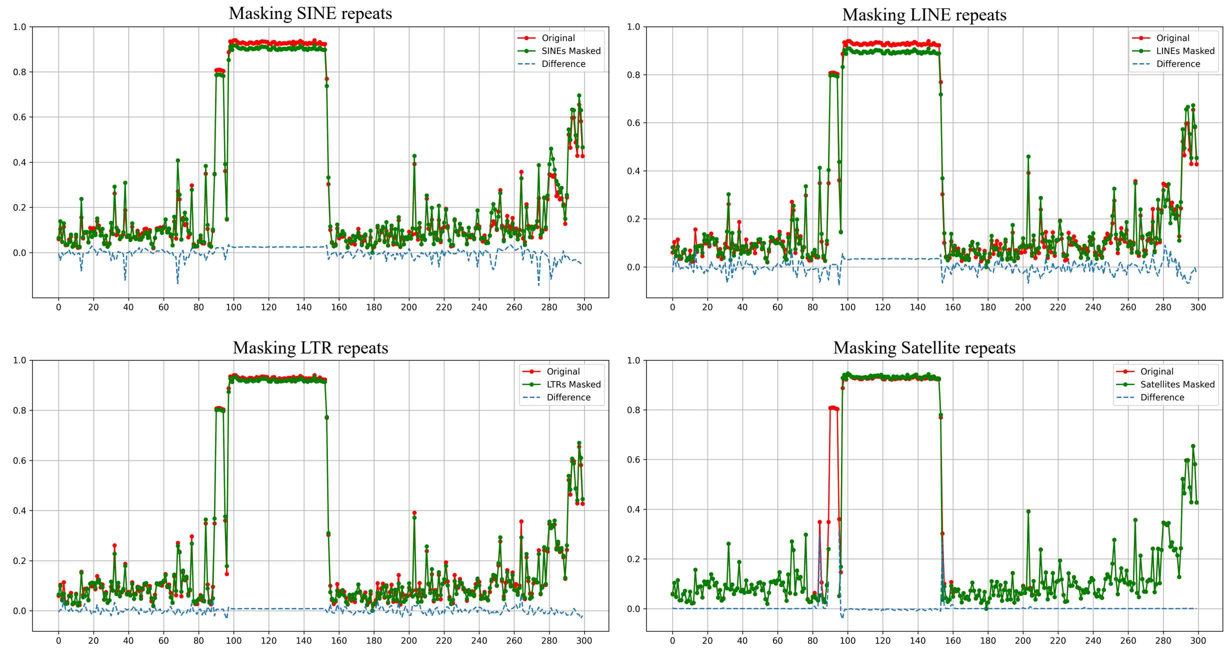

(b) Human chromosome 9.

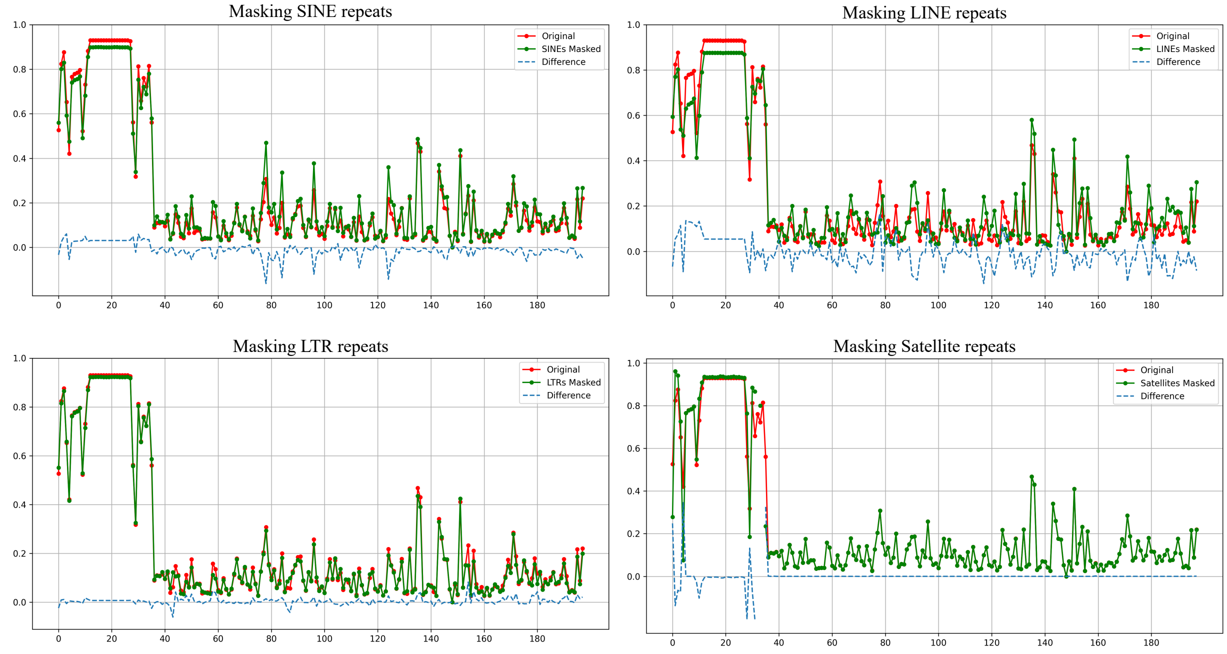

(c) Human chromosome 15.

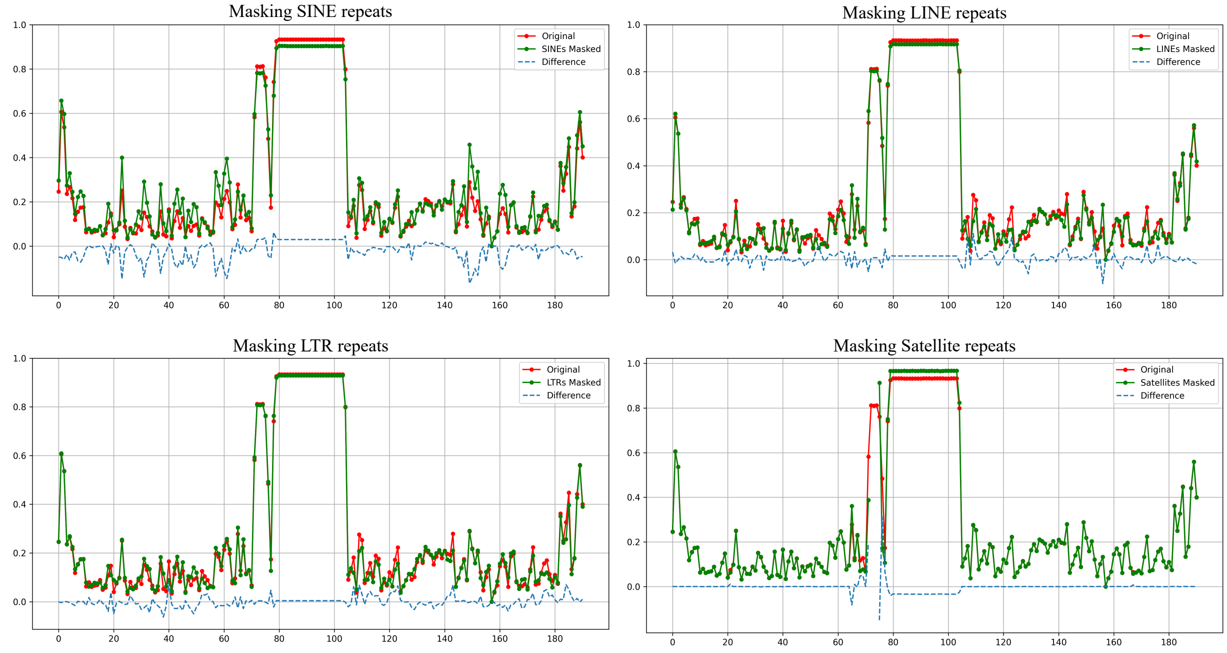

(d) Human chromosome 16.

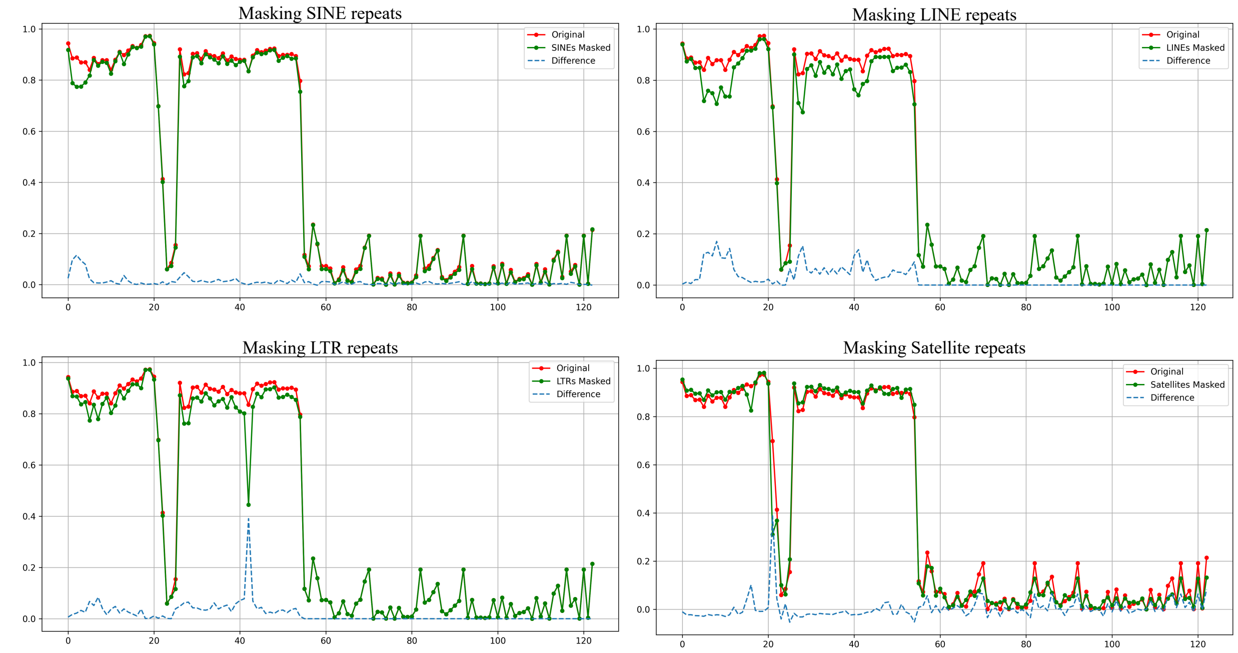

(e) Human chromosome Y.

Figure S7: **Effect of masking different repeat classes on intragenomic variation along human chromosome 1, 9, 15, 16, and Y.** In each subfigure, each panel plots the DSSIM distance between a chromosome-representative segment and each of consecutive 500 Kbp segments of the chromosome, as follows: (i) before masking (red), (ii) after masking (green), and (iii) their difference (dashed blue). In all experiments, the chromosome-representative segment is selected from the unmasked chromosome and kept fixed, to allow consistent comparison across masking conditions; repeat regions within this representative segment are also masked to match the masked genome. In some chromosomes, sometimes a discontinuity appears in "Masking Satellite repeats" plots (e.g., chromosome 16) because certain segments lie fully within the centromeric region and become entirely masked with 'N's, so the distance calculation is not applicable.

C Supplementary Tables

Table S1: Classification accuracy across k-mer sizes ($k = 3$–8) and sequence lengths (100 Kbp, 200 Kbp, 500 Kbp). The results show that $k = 4$–6 yield higher classification performance compared to other values. Among them, $k = 6$ was selected as the optimal setting, balancing accuracy and biological relevance. Sequence lengths of 200 Kbp and 500 Kbp achieve similar levels of accuracy and were used interchangeably in subsequent experiments.

Length (bp)	Representative Strategy	k					
		3	4	5	6	7	8
100,000	RepSeg	82.00	83.73	83.55	83.64	79.64	73.18
	aRepSeg	82.00	83.55	83.27	82.91	79.36	72.27
	Random	73.43	76.76	77.69	75.54	70.70	60.96
200,000	RepSeg	84.18	85.00	87.18	84.91	82.36	76.45
	aRepSeg	83.09	84.55	87.00	84.45	81.91	75.55
	Random	77.97	80.46	80.76	77.63	75.43	68.01
500,000	RepSeg	83.91	85.82	87.00	86.73	84.00	80.09
	aRepSeg	83.82	85.55	86.45	86.73	83.82	80.09
	Random	79.77	82.18	82.62	80.88	78.08	72.68

Table S2: Most frequent 9-mers across all human chromosomes, including their counts and percentages. The table reports the top three most frequent 9-mers identified in each human chromosome (1–22, X, and Y), based on their raw occurrence counts and the percentage each represents out of the total number of 9-mers in that chromosome. These values are shown alongside the total length of each chromosome. Notably, 9-mers such as TTTTTTTTT and AAAAAAAAA appear frequently across multiple chromosomes, while chromosomes 9 and Y exhibit exceptionally high occurrences of specific 9-mers, such as AAGGTAAGG and TAAGGTAAG, respectively. The **bolded** chromosomes indicate those with lighter FCGR images.

	First most frequent 9-mer			Second most frequent 9-mer			Third most frequent 9-mer			
Chromosome	9-mer	Count	Percentage (%)	9-mer	Count	Percentage (%)	9-mer	Count	Percentage (%)	Length
1	TAAGGTAAG	419,141	0.1687	CTAAGGTAA	370,733	0.1493	TTTTTTTTT	306,020	0.1232	248,387,328
2	TTTTTTTTT	284,426	0.1172	AAAAAAAAA	283,958	0.117	TGTGTGTGT	61,761	0.0254	242,696,752
3	TTTTTTTTT	234,251	0.1165	AAAAAAAAA	229,535	0.1141	TATAAAATA	50,278	0.025	201,105,948
4	AAAAAAAAA	200,030	0.1033	TTTTTTTTT	196,535	0.1015	TATATATAT	49,139	0.0254	193,574,945
5	TTTTTTTTT	203,980	0.112	AAAAAAAAA	201,762	0.1108	TGTGTGTGT	49,119	0.027	182,045,439
6	TTTTTTTTT	198,419	0.1153	AAAAAAAAA	197,183	0.1146	TGTGTGTGT	41,988	0.0244	172,126,628
7	TTTTTTTTT	208,597	0.1299	AAAAAAAAA	203,331	0.1266	TGTGTGTGT	38,961	0.0242	160,567,428
8	TTTTTTTTT	165,830	0.1134	AAAAAAAAA	163,248	0.1116	TGTGTGTGT	35,980	0.0246	146,259,331
**9**	AAGGTAAGG	1,134,132	0.753	TAAGGTAAG	1,125,559	0.7473	CCTTACCTT	1,122,261	0.7451	150,617,247
10	AAAAAAAAA	168,754	0.1252	TTTTTTTTT	168,639	0.1251	TAAGGTAAG	42,979	0.0319	134,758,134
11	AAAAAAAAA	154,443	0.1143	TTTTTTTTT	153,388	0.1135	TGTGTGTGT	32,287	0.0239	135,127,769
12	TTTTTTTTT	178,219	0.1337	AAAAAAAAA	177,253	0.133	ACACACACA	35,268	0.0265	133,324,548
13	TTTTTTTTT	113,729	0.1001	AAAAAAAAA	106,388	0.0937	TATAAAATA	101,637	0.0895	113,566,686
14	TTTTTTTTT	120,568	0.1192	AAAAAAAAA	117,474	0.1161	AGGTAAGGT	47,844	0.0473	101,161,492
**15**	CTTACCTTA	337,664	0.3385	TTACCTTAC	301,583	0.3023	ACCTTACCT	297,874	0.2986	99,753,195
**16**	TTACCTTAG	347,332	0.3606	CTTACCTTA	306,018	0.3177	TACCTTAGT	261,549	0.2715	96,330,374
17	AAAAAAAAA	150,399	0.1785	TTTTTTTTT	145,702	0.1729	GGTCCGACC	26,442	0.0314	84,276,897
18	AAAAAAAAA	83,308	0.1034	TTTTTTTTT	82,268	0.1021	AAAGACTCT	24,864	0.0309	80,542,538
19	TTTTTTTTT	129,281	0.2095	AAAAAAAAA	126,436	0.2049	GGTCGGACC	26,546	0.043	61,707,364
20	TTTTTTTTT	84,756	0.128	AAAAAAAAA	79,342	0.1198	TAAGGTAAG	64,072	0.0968	66,210,255
21	AGGTAAGGT	59,857	0.1327	TAAGGTAAG	58,526	0.1298	AAGGTAAGG	58,302	0.1293	45,090,682
22	AGGTAAGGT	78,190	0.1523	TAAGGTAAG	71,040	0.1384	AAGGTAAGG	69,545	0.1355	51,324,926
X	AAAAAAAAA	163,235	0.1058	TTTTTTTTT	162,823	0.1056	TATATATAT	44,877	0.0291	154,259,566
**Y**	TAAGGTAAG	624,052	0.9991	GTAAGGTAA	546,004	0.8742	AAGGTAAGG	524,222	0.8393	62,460,029

Table S3: Most frequent 9-mers across all maize chromosomes, including their counts and percentages. The table lists the top three most frequent 9-mers found in each maize chromosome (1–10), reporting both their raw counts and the percentage they consist of the total number of 9-mers within the respective chromosome. These values are shown alongside the total length of each chromosome. Notably, chromosomes 2 and 4 display particularly high occurrences of specific 9-mers. The **bolded** chromosomes indicate those with lighter FCGR images.

	First most frequent 9-mer			Second most frequent 9-mer			Third most frequent 9-mer			
Chromosome	9-mer	Count	Percentage (%)	9-mer	Count	Percentage (%)	9-mer	Count	Percentage (%)	Length
1	TCATCATCA	105,963	0.0345	CATCATCAT	104,841	0.0341	ATCATCATC	104,328	0.0339	307,335,809
**2**	TCATCATCA	246,147	0.0995	CATCATCAT	245,784	0.0993	ATCATCATC	245,548	0.0992	247,431,027
3	ACAGGCCAC	48,425	0.01994	GTGGCCTGT	48,384	0.0199	TATATATAT	40,761	0.0168	242,843,974
**4**	TGATGATGA	124,373	0.0495	GATGATGAT	120,650	0.0480	ATGATGATG	120,563	0.048	251,128,394
5	ACAGGCCAC	46,751	0.0212	GTGGCCTGT	46,086	0.0209	CGTGGCCTG	36,383	0.0165	220,303,002
6	ACAGGCCAC	37,512	0.0186	GTGGCCTGT	35,201	0.0174	CAGGCCACG	29,567	0.0147	201,729,004
7	GTGGCCTGT	35,043	0.0193	ACAGGCCAC	34,498	0.019	ATATATATA	30,878	0.017	181,266,306
8	TTTTATCGG	61,516	0.0295	TTTATCGGT	61,255	0.0294	TTATCGGTA	60,700	0.0291	208,583,295
9	ACAGGCCAC	32,683	0.0194	GTGGCCTGT	32,582	0.0194	ATATATATA	27,686	0.0165	168,156,889
10	ACAGGCCAC	28,545	0.0191	GTGGCCTGT	28,458	0.019	TATATATAT	28,038	0.0187	149,826,620

Table S4: The p-values from the Wilcoxon signed-rank test for various distance measures, comparing the human-human distances with the distances between human and each species in the corresponding row. It is expected that no significant difference (i.e., a p-value greater than 0.05) will be observed when comparing human-human distances with human-*P. troglodytes* and human-*M. musculus*, as they are both from the Mammalian class. However, the Manhattan, Descriptor, and Wasserstein distance measures yield values smaller than 0.05 when comparing human-human distances with human-*M. musculus*, which does not support the expectation of no significant difference among mammals.

human-human vs. human-*other species*	Normalized Euclidean	Cosine	Manhattan	Descriptor	DSSIM	LPIPS	K-S	Wasserstein
P. troglodytes	0.1507	0.4745	0.1648	0.2355	0.1777	0.3919	0.3348	0.9124
M. musculus	0.1161	0.6352	9.0e-4	0.0348	0.0961	0.9206	0.6799	0.0415
D. melanogaster	6.75e-10	4.67e-11	1.79e-11	6.99e-7	1.11e-14	6.00e-8	8.94e-16	0.0336
S. cerevisiae	1.10e-6	2.81e-8	5.01e-11	0.0256	3.54e-13	5.89e-8	9.27e-14	0.0333
A. thaliana	3.69e-6	2.17e-8	2.04e-7	0.0059	1.09e-11	3.40e-6	1.24e-9	0.0684
P. caudatum	3.44e-15	3.24e-14	5.19e-15	9.88e-4	1.53e-14	2.26e-16	1.38e-15	2.26e-16
P. furiosus	1.75e-16	3.42e-12	3.90e-18	9.87e-16	1.15e-17	6.54e-17	1.73e-8	2.07e-4
E. coli	1.74e-17	1.27e-15	3.90e-18	1.91e-16	4.27e-18	4.17e-15	1.16e-14	2.73e-6

Table S5: Effect of the hyperparameter n (the size of the set $\hat{S}$ in ARSSP) on runtime and mean absolute error (MAE) between ARSSP and RSSP for human chromosome 1. The MAE and execution time, averaged over 100 runs of the ARSSP pipeline, illustrate how changes in n influence both accuracy and computational cost. The time improvement, representing the relative speedup compared to the RSSP execution time (~100s), highlights the trade-off between computational efficiency and accuracy, which provides insights into the performance of ARSSP in comparison to RSSP.

n	MAE	Execution Time (s)	Time Improvement
50	0.025	9	11.11x
40	0.025	7	14.29x
30	0.027	5	20x
20	0.034	3	33.33x
10	0.045	1	100x
1	0.196	0.12	833.33x

Table S6: Quantitative summary of the effect of masking different repeat classes on human chromosome 1, 9, 15, 16, and Y. Each row corresponds to an experiment in which a specific repeat class was independently masked, and DSSIM distances were recalculated relative to the same chromosome-representative segment identified from the unmasked chromosome. Reported values include: chromosome name; repeat class; the total masked length (in base pairs) within the chromosom; the percentage of the chromosome-representative segment affected by masking; the number of segments whose distance from the chromosome-representative is below the DSSIM distance threshold of 0.24; the mean DSSIM distance across the chromosome; the mean squared error (MSE, range [0,1]) when comparing the masked and the unmasked intragenomic variation. The total numbers of 500 Kbp segments for each chromosome are: Chromosome 1 = 495, Chromosome 9 = 300, Chromosome 15 = 198, Chromosome 16 = 191, and Chromosome Y = 123.

Chromosome	Repeat class	Total Length (bp)	(%) in Representative	Segments < 0.24	Mean DSSIM	MSE
1	Unmasked	248,387,328	N/A	398	0.1975	N/A
	SINEs	36,491,829	13.37	359	0.2121	0.0016
	LINEs	55,190,608	28.33	415	0.1923	0.0016
	LTRs	23,051,659	12.09	401	0.1978	0.0003
	Satellites	19,029,251	0	398	0.1896	0.0003
9	Unmasked	150,617,247	N/A	209	0.2839	N/A
	SINEs	17,265,468	12.24	204	0.2861	0.0007
	LINEs	30,096,147	26.61	210	0.2804	0.0007
	LTRs	12,359,000	6.90	209	0.2814	0.0002
	Satellites	9,209,939	0	213	0.2722	0.0009
15	Unmasked	99,753,195	N/A	154	0.2310	N/A
	SINEs	13,690,275	15.90	147	0.2416	0.0011
	LINEs	20,770,470	34.23	142	0.2371	0.0036
	LTRs	7,845,559	5.99	155	0.2268	0.0002
	Satellites	6,507,451	0.01	157	0.2254	0.0022
16	Unmasked	96,330,374	N/A	140	0.2714	N/A
	SINEs	17,000,988	16.91	123	0.2922	0.0023
	LINEs	15,184,290	15.00	145	0.2647	0.0006
	LTRs	7,563,586	3.54	142	0.2709	0.0005
	Satellites	15,444,307	0	141	0.2650	0.0011
Y	Unmasked	62,460,029	N/A	71	0.4083	N/A
	SINEs	4,498,508	5.96	71	0.3977	0.0004
	LINEs	7,089,900	0	71	0.3837	0.0022
	LTRs	4,850,185	0	71	0.3901	0.0019
	Satellites	11,764,011	35.18	71	0.4046	0.0020

Table S7: Classification performance between the first chromosomes of human and chimpanzee after independently masking different repeat classes (SINEs, LINEs, LTRs, and Satellites). Accuracy, macro precision, and macro F1-score are reported for the unmasked and masked conditions.

Repeat class	Accuracy (%)	Macro Precision (%)	Macro F1 (%)
Unmasked	52	52.03	51.83
SINEs	49.75	49.72	48.30
LINEs	52.25	52.31	51.96
LTRs	49	48.94	48.32
Satellites	52	52.03	51.83

D NCBI Links for dataset

Table S8: NCBI genome assemblies and download links for the species used in this study.

Species	Assembly	Link
Homo sapiens (human)	GCA_009914755.4	https://www.ncbi.nlm.nih.gov/datasets/genome/GCA_009914755.4/
Pan troglodytes (chimpanzee)	GCA_028858775.2	https://www.ncbi.nlm.nih.gov/datasets/genome/GCA_028858775.2/
Mus musculus (house mouse)	GCA_000001635.9	https://www.ncbi.nlm.nih.gov/datasets/genome/GCA_000001635.9/
Drosophila melanogaster (fruit fly)	GCA_000001215.4	https://www.ncbi.nlm.nih.gov/datasets/genome/GCA_000001215.4/
Saccharomyces cerevisiae	GCA_000146045.2	https://www.ncbi.nlm.nih.gov/datasets/genome/GCA_000146045.2/
Arabidopsis thaliana (thale cress)	GCA_000001735.2	https://www.ncbi.nlm.nih.gov/datasets/genome/GCA_000001735.2/
Paramecium caudatum	GCA_000715435.1	https://www.ncbi.nlm.nih.gov/datasets/genome/GCA_000715435.1/
Pyrococcus furiosus	GCA_008245085.1	https://www.ncbi.nlm.nih.gov/datasets/genome/GCA_008245085.1/
Escherichia coli	GCA_000005845.2	https://www.ncbi.nlm.nih.gov/datasets/genome/GCA_000005845.2/
Aspergillus nidulans	GCA_000011425.1	https://www.ncbi.nlm.nih.gov/datasets/genome/GCA_000011425.1/
Zea mays (maize)	GCA_022117705.1	https://www.ncbi.nlm.nih.gov/datasets/genome/GCA_022117705.1/
Dictyostelium discoideum	GCA_000004695.1	https://www.ncbi.nlm.nih.gov/datasets/genome/GCF_000004695.1/

References

[1] Arvind Babu and Ram S. Verma. Chromosome structure: Euchromatin and heterochromatin. *International Review of Cytology*, 108(C):1–60, January 1987. https://doi.org/10.1016/S0074-7696(08)61435-7.

[2] Katrina F. Cooper, Richard B. Fisher, and Chris Tyler-Smith. Structure of the pericentric long arm region of the human Y chromosome. *Journal of Molecular Biology*, 228(2):421–432, 1992. https://doi.org/10.1016/0022-2836(92)90831-4.

[3] Jorge Duitama, Alena Zablotskaya, Rita Gemayel, An Jansen, Stefanie Belet, Joris Vermeesch, Kevin Verstrepen, and Guy Froyen. Large-scale analysis of tandem repeat variability in the human genome. *Nucleic Acids Research*, 42, 03 2014. https://doi.org/10.1093/nar/gku212.

[4] Stephen L. France, J. Douglas Carroll, and Hui Xiong. Distance metrics for high dimensional nearest neighborhood recovery: Compression and normalization. *Information Sciences*, 184(1):92–110, 2012. https://doi.org/10.1016/j.ins.2011.07.048.

[5] Uta Francke and Noëlynn Oliver. Quantitative analysis of high-resolution trypsin-Giemsa bands on human prometaphase chromosomes. *Human Genetics*, 45:137–165, 1978. https://doi.org/10.1007/BF00286957.

[6] Deborah L Grady, Robert L Ratliff, Donna L Robinson, Erin C McCanlies, Julianne Meyne, and Robert K Moyzis. Highly conserved repetitive DNA sequences are present at human centromeres. *Proceedings of the National Academy of Sciences*, 89(5):1695–1699, 1992. https://doi.org/10.1073/pnas.89.5.1695.

[7] Matthew B. Hufford, Arun S. Seetharam, Margaret R. Woodhouse, Kapeel M. Chougule, Shujun Ou, et al. De novo assembly, annotation, and comparative analysis of 26 diverse maize genomes. *Science*, 373:655–662, 08 2021. https://doi.org/10.1126/science.abg5289.

[8] Rallis Karamichalis, Lila Kari, Stavros Konstantinidis, and Steffen Kopecki. An investigation into inter- and intragenomic variations of graphic genomic signatures. *BMC Bioinformatics*, 16, 02 2015. https://doi.org/10.1186/s12859-015-0655-4.

[9] Svetlana Lazebnik, Cordelia Schmid, and J. Ponce. Beyond bags of features: Spatial pyramid matching for recognizing natural scene categories. In *Proceedings of the IEEE/CVF Conference on Computer Vision and Pattern Recognition (CVPR)*, volume 2, pages 2169–2178, 2006. https://doi.org/10.1109/CVPR.2006.68.

[10] Xingyu Liao, Wufei Zhu, Juexiao Zhou, Haoyang Li, Xiaopeng Xu, Bin Zhang, and Xin Gao. Repetitive DNA sequence detection and its role in the human genome. *Communications Biology*, 6(1):954, 2023. https://doi.org/10.1038/s42003-023-05322-y.

[11] Kah-Wai Lin and Ju Yan. The telomere length dynamic and methods of its assessment. *Journal of Cellular and Molecular Medicine*, 9(4):977–989, 2005. https://doi.org/10.1111/j.1582-4934.2005.tb00395.x.

[12] Chunjie Luo, Jianfeng Zhan, Xiaohe Xue, Lei Wang, Rui Ren, and Qiang Yang. Cosine normalization: Using cosine similarity instead of dot product in neural networks. In Věra Kůrková, Yannis Manolopoulos, Barbara Hammer, Lazaros Iliadis, and Ilias Maglogiannis, editors, *Artificial Neural Networks and Machine Learning – ICANN 2018*, pages 382–391, Cham, 2018. Springer International Publishing. https://doi.org/10.1007/978-3-030-01418-6_38.

[13] Brian McStay. The p-arms of human acrocentric chromosomes play by a different set of rules. *Annual Review of Genomics and Human Genetics*, 24, 02 2022. https://doi.org/10.1146/annurev-genom-101122-081642.

[14] Olivia Morrison and Jitendra Thakur. Molecular complexes at euchromatin, heterochromatin and centromeric chromatin. *International Journal of Molecular Sciences*, 22(13), 2021. https://doi.org/10.3390/ijms22136922.

[15] NCBI Genome Data Viewer. NCBI Genome Browser: GCF_902167145.1, 2024. Available at: https://www.ncbi.nlm.nih.gov/gdv/browser/genome/?id=GCF_902167145.1.

[16] Jamy C. Peng and Gary H. Karpen. Heterochromatic genome stability requires regulators of histone H3 K9 methylation. *PLoS Genetics*, 5(3):e1000435, 2009. https://doi.org/10.1371/journal.pgen.1000435.

[17] RepeatMasker Open-4.0. RepeatMasker, 2013. Smit, AFA, Hubley, R & Green, P. RepeatMasker Open-4.0. 2013–2015. Available at: http://www.repeatmasker.org.

[18] Arang Rhie, Sergey Nurk, Monika Cechova, Savannah J. Hoyt, Dylan J. Taylor, Nicolas Altemose, Paul W. Hook, Sergey Koren, et al. The complete sequence of a human Y chromosome. *Nature*, 621, 08 2023. https://doi.org/10.1038/s41586-023-06457-y.

[19] Nalini Srinivas, Sivaramakrishna Rachakonda, and Rajiv Kumar. Telomeres and telomere length: A general overview. *Cancers*, 12(3):558, 2020. https://doi.org/10.3390/cancers12030558.

[20] Kaitlin M. Stimpson, Ihn Young Song, Anna Jauch, Heidi Holtgreve-Grez, Karen E. Hayden, Joanna M. Bridger, and Beth A. Sullivan. Telomere disruption results in non-random formation of de novo dicentric chromosomes involving acrocentric human chromosomes. *PLoS Genetics*, 6(8):1–19, 08 2010. https://doi.org/10.1371/journal.pgen.1001061.

[21] Yuta Suzuki and Shinichi Morishita. The time is ripe to investigate human centromeres by long-read sequencing†. *DNA Research*, 28(6):dsab021, 10 2021. https://doi.org/10.1093/dnares/dsab021.

[22] Hisashi Tamaru. Confining euchromatin/heterochromatin territory: jumonji crosses the line. *Genes & Development*, 24(14):1465–1478, 2010. https://doi.org/10.1101/gad.1941010.

[23] Zhou Wang, Alan Bovik, Hamid Sheikh, and Eero Simoncelli. Image quality assessment: From error visibility to structural similarity. *IEEE Transactions on Image Processing*, 13:600 – 612, 05 2004. https://doi.org/10.1109/TIP.2003.819861.

[24] Peter Warburton, Dan Hasson, Flavia Guillem, Chloé Lescale, Xiaoping Jin, and Gyorgy Abrusan. Analysis of the largest tandemly repeated DNA families in the human genome. *BMC Genomics*, 9:533, 12 2008. https://doi.org/10.1186/1471-2164-9-533.

[25] Kurt Whittemore, Elsa Vera, Eva Martínez-Nevado, Carola Sanpera, and Maria A Blasco. Telomere shortening rate predicts species life span. *Proceedings of the National Academy of Sciences*, 116(30):15122–15127, 2019. https://doi.org/10.1073/pnas.1902452116.

[26] Richard Zhang, Phillip Isola, Alexei A. Efros, Eli Shechtman, and Oliver Wang. The unreasonable effectiveness of deep features as a perceptual metric. In *Proceedings of the IEEE/CVF Conference on Computer Vision and Pattern Recognition (CVPR)*, pages 586–595, 2018. https://doi.org/10.1109/CVPR.2018.00068.
